# An artificial chromosome for data storage

**DOI:** 10.1093/nsr/nwab028

**Published:** 2021-02-12

**Authors:** Weigang Chen, Mingzhe Han, Jianting Zhou, Qi Ge, Panpan Wang, Xinchen Zhang, Siyu Zhu, Lifu Song, Yingjin Yuan

**Affiliations:** School of Microelectronics, Tianjin University, Tianjin 300072, China; Frontier Science Center for Synthetic Biology and Key Laboratory of Systems Bioengineering (Ministry of Education), Tianjin University, Tianjin 300072, China; Frontier Science Center for Synthetic Biology and Key Laboratory of Systems Bioengineering (Ministry of Education), Tianjin University, Tianjin 300072, China; SynBio Research Platform, Collaborative Innovation Center of Chemical Science and Engineering (Tianjin), School of Chemical Engineering and Technology, Tianjin University, Tianjin 300072, China; Frontier Science Center for Synthetic Biology and Key Laboratory of Systems Bioengineering (Ministry of Education), Tianjin University, Tianjin 300072, China; SynBio Research Platform, Collaborative Innovation Center of Chemical Science and Engineering (Tianjin), School of Chemical Engineering and Technology, Tianjin University, Tianjin 300072, China; School of Microelectronics, Tianjin University, Tianjin 300072, China; School of Microelectronics, Tianjin University, Tianjin 300072, China; Frontier Science Center for Synthetic Biology and Key Laboratory of Systems Bioengineering (Ministry of Education), Tianjin University, Tianjin 300072, China; SynBio Research Platform, Collaborative Innovation Center of Chemical Science and Engineering (Tianjin), School of Chemical Engineering and Technology, Tianjin University, Tianjin 300072, China; Frontier Science Center for Synthetic Biology and Key Laboratory of Systems Bioengineering (Ministry of Education), Tianjin University, Tianjin 300072, China; SynBio Research Platform, Collaborative Innovation Center of Chemical Science and Engineering (Tianjin), School of Chemical Engineering and Technology, Tianjin University, Tianjin 300072, China; Frontier Science Center for Synthetic Biology and Key Laboratory of Systems Bioengineering (Ministry of Education), Tianjin University, Tianjin 300072, China; SynBio Research Platform, Collaborative Innovation Center of Chemical Science and Engineering (Tianjin), School of Chemical Engineering and Technology, Tianjin University, Tianjin 300072, China; Frontier Science Center for Synthetic Biology and Key Laboratory of Systems Bioengineering (Ministry of Education), Tianjin University, Tianjin 300072, China; SynBio Research Platform, Collaborative Innovation Center of Chemical Science and Engineering (Tianjin), School of Chemical Engineering and Technology, Tianjin University, Tianjin 300072, China

**Keywords:** DNA storage, synthetic biology, indel correction, encoded DNA, artificial chromosome

## Abstract

DNA digital storage provides an alternative for information storage with high density and long-term stability. Here, we report the *de novo* design and synthesis of an artificial chromosome that encodes two pictures and a video clip. The encoding paradigm utilizing the superposition of sparsified error correction codewords and pseudo-random sequences tolerates base insertions/deletions and is well suited to error-prone nanopore sequencing for data retrieval. The entire 254 kb sequence was 95.27% occupied by encoded data. The Transformation-Associated Recombination method was used in the construction of this chromosome from DNA fragments and necessary autonomous replication sequences. The stability was demonstrated by transmitting the data-carrying chromosome to the 100th generation. This study demonstrates a data storage method using encoded artificial chromosomes via *in vivo* assembly for write-once and stable replication for multiple retrievals, similar to a compact disc, with potential in economically massive data distribution.

## Teaser

A 254 kb artificial chromosome, storing two pictures and one video clip encoded with low-density parity-check codes and pseudo-random sequences, was manufactured and transmitted from generation to generation stably. The digital information residing in the chromosome was fast retrieved, tolerating base insertions and deletions in nanopore readout.

## INTRODUCTION

Rapid progress in synthetic biology during the last two decades has provided powerful tools for the design and chemical synthesis of genomic DNAs with specific functions as desired [1,2]. Examples include genomic DNAs from *Escherichia coli* [3], *Saccharomyces cerevisiae* [4–6] and *Mycoplasma mycoides* [7] etc*.* Recently, a number of studies demonstrated the possibility of using DNA to store digital information instead of genetic information [8–22]. This prompted us to seek the possibility of design and synthesis of a chromosome fully dedicated to information storage.

With the development of high-throughput DNA synthesis and sequencing technologies, large-scale data storage in DNA has become feasible [9–14]. Presently, oligo-based efforts are limited by the non-uniformity of *in vitro* DNA amplification efficiency [14]. Artificial chromosomes introduced to live cells can self-replicate with high accuracy and low cost, which could represent a practical trend in archival storage. The idea of information storage in live cells through DNA has a long history [23]. A recent success of storing a digital movie in a population of bacteria was reported [24]. The lengths of artificial DNAs in live cells for the purpose of information storage were summarized in Table S1 and have never exceeded several thousand bases per cell [23–31].

In this study, we design and synthesize a yeast artificial chromosome (YAC) containing 254 886 bp using methods previously reported [4,5,32], allowing us to perform an in-depth evaluation of the stability of large-data-encoded DNA. Two pictures and a video clip were encoded in this chromosome using a superposition coding scheme. The stability of this artificial chromosome during yeast replication was well maintained through serial batch cultivation. *In vivo* assembly of the encoded artificial chromosome is analogous to burning a CD, which is a write-once action, while stable replications of the chromosome allow CD-like multiple retrievals. We thus proved the feasibility of a data storage paradigm using an artificial chromosome with a specialized encoding system.

## RESULTS

**Figure 1.**
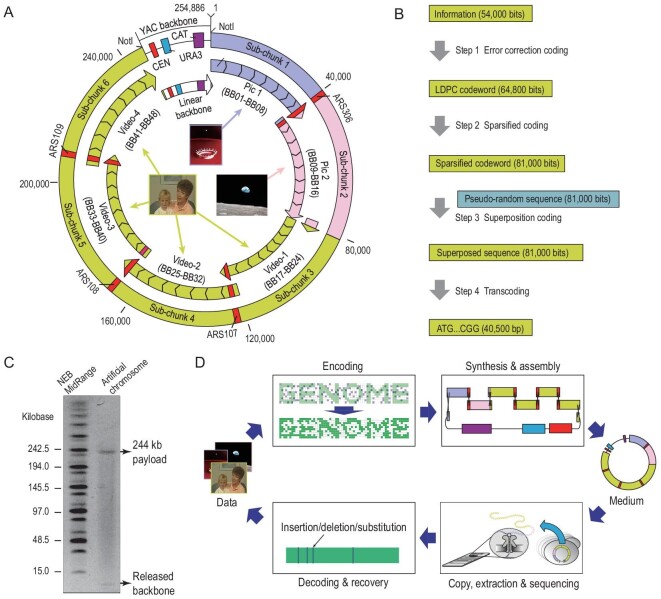
Design and assembly of a data-carrying artificial chromosome. (A) Schematic diagram of the data-carrying chromosome. Four additional ARSs were inserted at specific positions as labeled, and BB is short for building block. (B) The encoding scheme. Superposition coding with LDPC codes (*R *= 5/6) and pseudo-random sequences converted information sub-chunks (54 000 bits) into the DNA sequence (40 500 bp). The design is detailed in Note S1. (C) The NotI digestion of the artificial chromosome released two bands. Payload bands, 244 kb; backbone band, 10 kb. (D) The workflow of this digital data storage mode.

A YAC of 254 886 bp, specialized for data storage, of which 95.27% was data payload, was designed and constructed as shown in Figs 1A and S1. Sparsified low-density parity-check (LDPC) codes and pseudo-random sequences are superposed to convert two pictures and a video into DNA sequences (Fig. 1B). This artificial chromosome was assembled from six DNA chunks with four autonomously replicating sequences (ARSs) to stabilize the replication. Success in construction was shown by pulsed-field gel electrophoresis (PFGE) (Fig. 1C). A portable MinION sequencer from Oxford Nanopore Technologies (ONT) was employed for rapid retrieval of the encoded data. Although noisy long reads were produced, the original files can be retrieved reliably (Figs 1D and S2).

### Superposition coding for chromosome-based DNA storage

**Figure 2.**
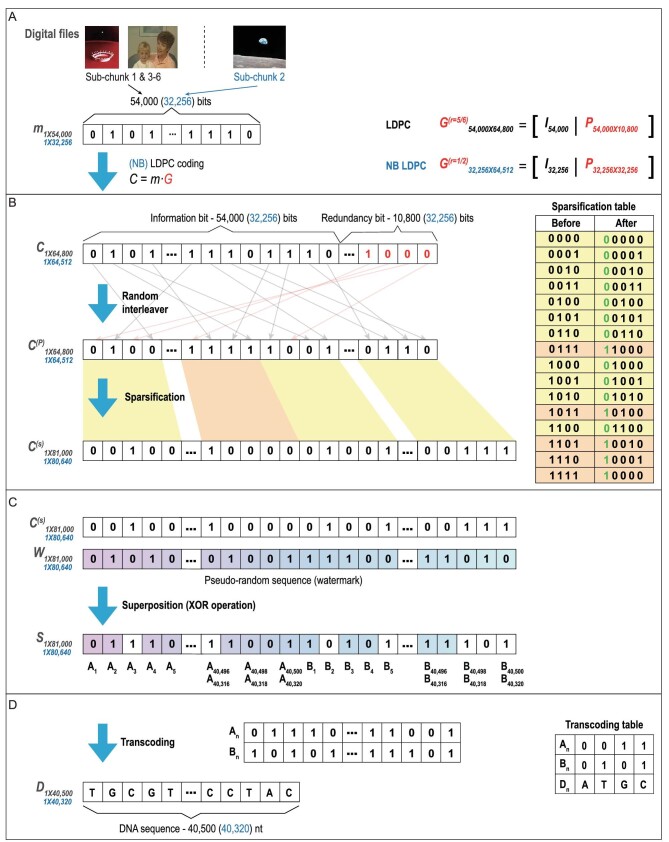
Encoding scheme for chromosome-based DNA storage. (A) Error correction coding. Digital files were divided into bit blocks, denoted as *m*, which was multiplied by generator matrix *G* for LDPC and NB LDPC codewords. (B) Sparsification of codewords. The codewords were randomly interleaved. The interleaved codewords were then sparsified by converting every 4 to 5 bits according to the sparsification table. (C) The sparsified codewords were superposed with a predetermined pseudo-random sequence (called watermark) by exclusive or XOR operation. (D) The DNA sequence was obtained by transcoding every 2 bits to 1 base according to the transcoding table.

A strategy of information coding, involving the superposition of sparsified LDPC codes and pseudo-random sequences, was chosen for this study (Fig. 2). LDPC codes are efficient block error correction codes, widely used in communications and data storage [33–35]. In our design, both binary and non-binary (NB) LDPC codes were employed (Fig. 2A). Digital files were divided into fixed-length blocks (54 000 bits for binary LDPC code and 32 256 bits for NB LDPC code). An interleaving step was introduced following LDPC coding in order to handle possible missing segments. The interleaved LDPC codewords were sparsified by mapping 4 to 5 bits (Fig. 2B) and superposed at the bit level with several carefully chosen pseudo-random sequences (called watermarks) (Fig. 2C). These pseudo-random sequences were used for indel identification and addressing, similar to the function of hidden hints in a jigsaw puzzle. Data DNA sequences were derived by transcoding (Fig. 2D), and integrated with the vector and ARSs to form a full artificial chromosome (Note S1, Fig. S3). A toy example of encoding 20 bits was illustrated in Fig. S4.

Using the aforementioned methods, we encoded 37 782-byte digital data, including two pictures and one video clip, into the artificial chromosome with a length of 254 886 bp including the YAC backbone and additional ARSs (Fig. 1A). The overall logical density (including YAC backbone) of this artificial chromosome is 1.19 bit/bp, which is similar to that of DNA Fountain (Table S2), as calculated in Note S2.

### Rationale for additional ARSs: key for 50% GC-content DNA assembly

**Figure 3.**
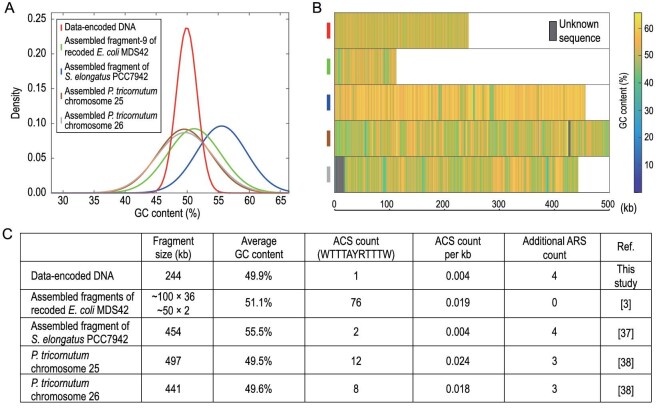
Rationale for ARSs added to the artificial chromosome. (A) Comparison of the GC contents in different assemblies. The DNA sequence as labeled was fragmented into 300 bp, and the GC content of each fragment was calculated. The number of fragments corresponding to different GC contents was normalized by the total fragment number and plotted. (B) Comparison of the maps of different assemblies showing the distribution of various GC contents. (C) Comparison of ACS counts in different assemblies.

The transcoding rule that we used (Fig. 2D) resulted in ∼50% guanine-cytosine (GC) content throughout the chromosome (Fig. 3A), and a GC distribution pattern different from genetically encoded YACs (Fig. 3B). Previous studies have reported that added ARSs could raise the assembly efficiency of an artificial chromosome and its stability [36–38]. Our data-encoded DNA contains only one yeast ARS consensus sequence (ACS, WTTTAYRTTTW) (Fig. 3C). In comparison with previously assembled YACs [3,37,38], the ACS count per kb was 0.004, equal to an assembly from *Synechococcus elongatus* PCC7942. We thus added four ARSs accordingly (Fig. 3C). As a result, the 254 886 bp data-carrying chromosome (pHM059) was assembled from six DNA chunks, 40 kb in length each, with additional four ARSs and a pCC1-Ura YAC backbone using the Transformation-Associated Recombination (TAR) method [39]. The rate of correct assembly was 9.4% (9 of 96 clones). Control experiments with no additional ARSs resulted in zero success rate.

### The storage-specific chromosome can be replicated stably with high fidelity

**Figure 4.**
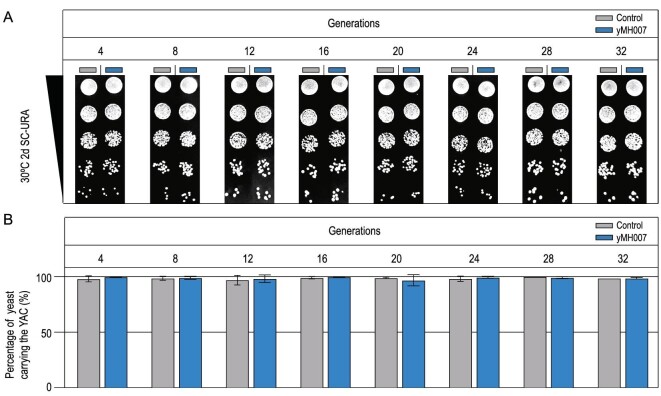
Analyses of the growth effect and stability of the data-carrying chromosome. (A) The effect of the artificial chromosome on the growth of the host. Yeast strains yMH007 and yMH104 (control) were serially diluted and spotted on the agar plate for growth. Cells were passed for different generations as indicated in liquid media before the assay. (B) Stability of the data-carrying chromosome. Same number of cells passed for different generations as indicated were sprayed on the SC-Ura and 5'-FOA plates. The colonies on both plates were calculated and their ratios were presented. The results were representative of three independent biological experiments with the corresponding standard deviations.

To test whether the data-carrying chromosome could be stably replicated in yeast, we cultured the strain yMH007 that harbors the data-carrying artificial chromosome and yMH104 that harbors an empty YAC backbone as control in liquid Synthetic Complete media without Uracil (SC-Ura). The growth rates of yMH007 and yMH104 were comparable, with doubling time being 2.7 ± 0.1 and 2.6 ± 0.1 hours (α = 0.05), respectively (Fig. S5A). Both strains were repetitively cultured for four generations (OD_600_ equal to 0.1 to 1.6) in fresh media before harvested for the next experiments. Serial dilutions of cells from various generations were spotted on an SC-Ura agar plate. The results showed that yeast harboring the encoded artificial chromosome grew as robustly as the control at 30°C (Fig. 4A). Next, we quantitated the colonies on SC-Ura and 5′-FOA plates, representing the number of cells that had maintained and lost the chromosome, respectively. Their ratios were similar to those of the controls, approximating 100% from all tested generations (Figs 4B and S5B). Passages of both strains in non-selective SC media were carried out, and the result showed that the data-carrying chromosome was gradually lost in the population as usual (Fig. S6).

BLAST search of the data-carrying DNA sequence against the National Center for Biotechnology Information (NCBI) nucleotide database revealed no homologous sequences. Interestingly, our preliminary data suggested that transcriptions on this artificial chromosome were active and that none of the 36 802 peptides detected by the data-independent acquisition (DIA) technique were coded by this chromosome. The transcriptional and translational profiles of pHM059 and its physiological impacts on the host are under further investigation in our laboratory.

The fidelity of the replication of the artificial chromosome was systematically assessed. Multiplex colony polymerase chain reactions (PCRs) were carried out using 96 clones with 20-generation intervals (20–100th, 12 clones each interval, 48 for the 100th). All bands with expected sizes could be observed on the agarose gel (Fig. S7A). The integrity of the chromosome extracted from 12 clones of the 100th generation was also evident by PFGE (Fig. S7B). Furthermore, high-throughput sequencing on Illumina HiSeq platform generated a dataset but detected no single mutation in the information chunk in any of the tested 24 samples from clones with 20-generation intervals (20–100th, three clones each interval, 12 for the 100th), consistent with a low mutation rate of yeast replication [40]. Taken together, we can safely conclude that the encoded artificial chromosome could be stably transmitted through 100 generations in selective media (SC-Ura), which is suitable for reliable information retrievals.

### Fast recovery from noisy nanopore readout

**Figure 5.**
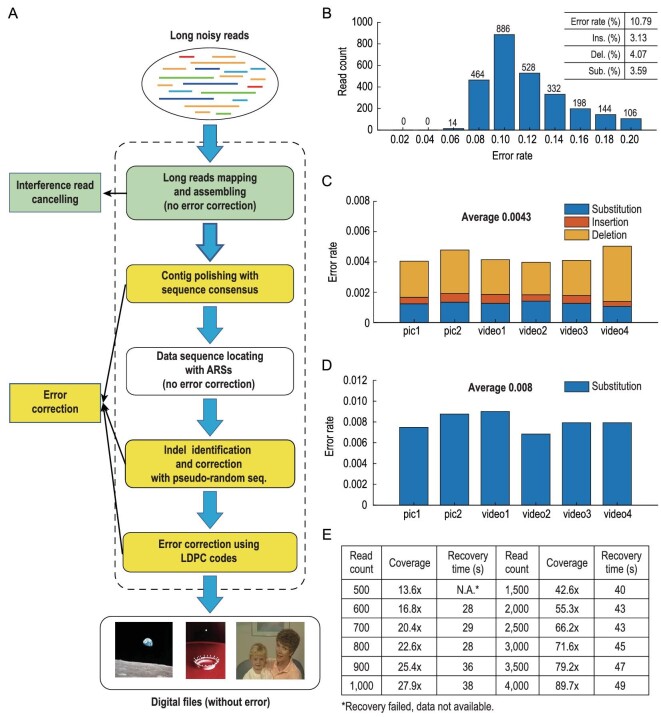
Error processing and data recovery. (A) Schematic flow of data recovery from nanopore sequencing reads. (B) Error rate distribution of reads from the artificial chromosome. Inset: error rate distribution by error typing. (C) Error rate distributions among six information sub-chunks after RACON polishing. Types of errors are labeled in different colors as indicated. (D) The distributions of substitution error rates among six information sub-chunks after indel identification and correction. (E) Recovery tests with various reads.

The third-generation MinION sequencer was used for the attempt to retrieve the files from the artificial chromosome, potentiated by its fastness, portability and capability of long sequencing. Upon the extraction of the artificial chromosome and library preparation, long raw reads were generated by the MinION sequencer with flow cell R9.4.1 within 10 min. The recovery process from the initial noisy reads is presented in Figs 5A and S8. The raw error rate was 10.79% (Fig. 5B). A stepwise assembly and polishing process was then performed to gradually lower the error rate. Briefly, traditional read-to-read overlap detection and the overlap-layout-consensus (OLC) assembly were first carried out, using Minimap and Miniasm, tools commonly used in fast mapping and *de novo* genome assembly [41]. These tools have no error correction functions and thus the assembled contigs still contained many errors, especially insertions and deletions. Next, we polished coarsely assembled contigs by Rapid Consensus (RACON) program [42]. With assembly and polishing, the error rate was reduced by an order of magnitude (Fig. 5C), and interference reads were also excluded (Fig. S9). Data DNA sequences were then located and extracted based on positioning ARSs and vector sequences (Fig. S10). Insertions and deletions were identified using modified forward–backward algorithms according to these superposed pseudo-random sequences [43] and then converted into substitution errors or erasures (Fig. 5D, Note S4), which were then corrected using LDPC codes in the final step [33–35]. We rapidly recovered the original files on a laptop computer (Ubuntu 16, Intel^®^ core™ i7–8565U, 16 GB RAM) within 40 seconds (Movie S1). The minimal coverage for data recovery was tested with various numbers of reads ranging from 500 to 4000 using a desktop computer (Intel^®^ core™ i9-9900K CPU @ 3.60 GHz, 128 GB RAM) (Fig. 5E). Results indicated that minimally 16.8 × coverage, equal to 600 reads, was enough for the recovery.

## DISCUSSION

In summary, we designed and synthesized an artificial chromosome consisting of 254 886 bp, carrying data that can be reliably retrieved from noisy nanopore reads. We also demonstrated the analogy of its data storage mode to CDs, regarding write-once and multiple retrievals. In addition, the information carried by our artificial chromosome can be massively copied with low cost due to faithful DNA self-replications in live cells. Our results also demonstrated that a portable and efficient nanopore-based reading device for information retrievals from an artificial chromosome is of great potential. Currently, however, the write-once process involving *in vitro* chemical synthesis of DNA and *in vivo* chromosome assembly is still expensive, and thus continuous reduction in writing cost remains a primary concern in the field of DNA archival storage.

Information storage using artificial chromosomes and oligo pools were compared with each other in Fig. S11. The advantages of using artificial chromosomes in information storage include less bias (Fig. S12A) and lower error rate (Fig. S12B) and cost per copy as the data retrieval process from the artificial chromosome is PCR-independent. Nanopore-based reading is also faster given that the encoding method we developed tolerates errors arising from nanopore sequencing. Our coding strategy is also compatible with Illumina-based sequencing (Fig. S13), which results in lower error rates (Table S4) but is more time-consuming.

It is of great importance to note the balance between coding density and other properties [43]. For example, a robust coding system with more redundancy sacrifices information density but tolerates error-prone faster reading, and a GC-content flexible coding system sacrifices information density but improves the stability of the artificial chromosome.

We envision that multiple artificial chromosomes in live cells, all dedicated to information storage, are practically doable, given that watermark-aided data retrievals can be performed in parallel. Such parallel readouts from two chromosomes (one being real, and the other being virtual) were simulated in Fig. S14.

## Methods

### Design of the artificial chromosome for digital data storage

We designed an artificial chromosome consisting of biological chunks and information chunks dedicated for digital data storage. The biological chunks included a YAC backbone and four additional ARSs to stabilize the artificial chromosome (Figs 1A and S1). Of the six information chunks, binary LDPC codes were used in five for the coding of a video clip and a picture, while NB LDPC codes were used in the remaining one to store a picture (Table S3). In addition to LDPC/NB LDPC coding, other strategies including interleaving, sparsification, superposition with watermarks, and transcoding are detailed in Note S1.

### Workflow of digital data storage using an artificial chromosome

The workflow of the digital storage with the artificial chromosome is divided into four steps (Figs 1B and S2). First, digital files (two pictures and a video) were mapped into data DNA sequences using the superposition coding method. Second, each data DNA sequence was decomposed into a series of sub-chunks with overlaps and outsourced for synthesis. The data DNA sequences were then assembled with additional ARSs and a YAC backbone. Third, the data-carrying chromosome was exponentially copied, extracted and sequenced. Fourth, following the proposed recovery process, digital files were fast retrieved using raw reads from the ONT MinION sequencer.

### Artificial chromosome stability assays in yeast

Baker yeast *S.cerevisiae* strains yMH007 and yMH104 were cultivated in flasks containing SC-Ura liquid medium in a shaker incubator at 30°C at 200 rpm. The overnight cultures were then diluted in a fresh SC-Ura liquid medium until OD_600_ equal to 0.1, and incubated at 30°C, until OD_600_ reached 1.6. Re-dilution and re-cultivation were repetitively carried out for the passage of generations. 50 μL of serially diluted cultures were spread on SC-Ura and SC supplemented with 5′-FOA agar plates. The colony numbers on both plates formed from cells with four-generation intervals were counted. The rate of chromosome-containing cells was calculated as *Num*_SC-Ura_/(*Num*_Sc-Ura _+ *Num*_SC+5-FOA_).

Yeast strain yMH007 was continuously cultivated to the 100th generation in SC-Ura medium at 30°C. Yeast colony multiplex PCRs were carried out using colonies formed by yeast cells with 20-generation intervals and primer sets 17–24 (Table S6).

## DATA AVAILABILITY

The data underlying this article will be shared on reasonable request to the corresponding author.

## CODE AVAILABILITY

The code is available from the corresponding author upon reasonable request.

## Supplementary Material

nwab028_Supplemental_File
