# Supplementary material for: An artificial chromosome for data storage
Source: Natl Sci Rev. 2021 Feb 12;8(5):nwab028. doi: 10.1093/nsr/nwab028 (PMC8288405; doi:10.1093/nsr/nwab028)
Supplement: nwab028_Supplemental_File [file nwab028_supplemental_file.docx]

**An artificial chromosome for data storage**

**Supplementary Materials**

Weigang Chen^†^, Mingzhe Han^†^, Jianting Zhou^†^, Qi Ge, Panpan Wang, Xinchen Zhang, Siyu Zhu, Lifu Song, Yingjin Yuan^*^

^†^These authors contributed equally to this work

^*^Corresponding author. Email: yjyuan@tju.edu.cn

Supplementary materials include:

Supplementary Note

Figure S1-S14

Table S1-S7

Movie S1

**Supplementary Note**

1. **Design details of the artificial chromosome**

The artificial chromosome consisted of the biological chunk and the information chunk (Fig. 1A). The biological chunk including a shuttle vector and four additional ARSs. The information chunk was generated using the superposition of sparsified error correction codewords and pseudo-random sequences.

**1.1 The biological chunk**

We constructed a YAC-BAC (pCC1-Ura) backbone reference to [1] as a shuttle vector between yeast and *E.coli* (Fig. S1). To guarantee the stability of the artificial chromosome, we embedded four additional ARSs (autonomously replicating sequences) between the adjacent two data sequences.

**1.2 The large information chunk**

Two types of information sub-chunks were embedded in the large information chunk. Five information sub-chunks were encoded with binary LDPC codes. The encoding procedure is shown in Fig. 1B and Fig. 2. A picture (milkcoronet.jpg, 6.5 KB) and a video clip (motheranddaughter.mp4, 25.5 KB) were encoded into five codewords, and each **has** a length of 64,800 bits. Then, the codewords were interleaved, sparsified, and superposed with pseudo-random sequences to form the binary sequences of 81,000 bits. Finally, binary sequences were converted into the information sub-chunks of 40,500 bp for each one. The other one information sub-chunk was encoded with non-binary LDPC codes. The encoding process is illustrated in Fig. S4. This picture (Earth_rise.jpg) has 4,029 bytes and we padded 3 bytes to obtain 4,032 bytes, i.e., 4,032×8=32,256 bits. Similarly, the original picture (Earthrise.jpg) was finally converted into a 40,320-bp information sub-chunk.

1. **Data payload ratio and logical density**

Data payload (Fig. S1) is the DNA chunks storing digital information. The ratio of data payload is 95.27%, which is defined as:

$$R=(Total length of data payload)/(Length of the YAC)=(40,500 bp\times5+40,320 bp)/(254,886 bp)\times100\%=95.27\%$$

The logical density (including YAC vector, *LD_i_*) can be calculated as [2]:

$${LD}_{i}=\frac{Total data stored}{Length of the YAC}=\frac{54,000 bits\times5+32,256 bits}{254,886 bp}=1.19 bit/bp$$

The logical density (excluding YAC vector, *LD_e_*) can be calculated as [2]:

$${LD}_{e}=\frac{Total data stored}{Data payload}=\frac{54,000 bits\times5+32,256 bits}{(40,500 bp\times5+40,320 bp)}=1.24 bit/bp$$

The comparison of logical density is listed in Table S2.

1. **Specification of superposition coding method**

**(1) LDPC codes**

LDPC codes are a type of block codes, whose error correction capability usually improves with the code length [3,4]. The decoding of LDPC codes is based on the parallel iterative algorithms, belief propagation algorithms, which can well adapt to the high throughput requirements of data storage using large DNA [5]. The iterative decoding complexity of LDPC codes increases linearly with the code length [6]. Therefore, we chose LDPC codes with large code length in this work. In order to evaluate the different error correction capabilities, we used two code rates, that is, 1/2 and 5/6, for two different sub-chunks.

**(2) Interleaving the LDPC codewords**

The LDPC codewords were interleaved by random permutation of all bits. The functions include: 1) Scattering the burst error (many errors occurring in a small fraction) into distributive random errors and facilitating the error correction [7]; 2) Avoiding long successive ‘0’s or ‘1’s, which may be converted into long DNA homopolymers.

**(3) Sparsified coding**

The interleaved LDPC codewords were sparsified by a mapping according to the sparsification table (Fig. 2). This can reduce the occurrence of bit “1”, preparing for the superposition with pseudo-random sequences (watermark).

**(4) Pseudo-random sequences (watermark)**

Pseudo-random sequences (watermark) were a bit-by-bit XOR of two different *m*-sequences [8, 9]. The functions of pseudo-random sequences include: 1) discrimination of the different data sequences from each other and host genome by different watermarks; 2) identification of indels [10] with the modified forward-backward algorithm (Note S4).

1. **Modified forward-backward algorithm for insertion/deletion identification**

A modified forward-backward algorithm based on the hidden Markov model (HMM) [11,12] is proposed to identify and correct the insertions and deletions with pseudo-random sequences (watermark).

**Parameter specification**

Define the state $x_{j}$ as the drift from the correct position index $j$. Define $I$ as the maximum number of consecutive insertion errors.

The state transition probability $P_{a,b}=P(x_{j+1}=b|x_{j}=a)$ is represented as [11]:

$$P_{a,b}=\left\{\begin{matrix} P_{d}, & b=a-1 \\ \alpha_{I}P_{i}P_{d}+P_{t}, & b=a \\ \alpha_{I}(P_{i})^{b-a+1}P_{d}+\alpha_{I}(P_{i})^{b-a}P_{t}, & a<b<a+I \\ \alpha_{I}(P_{i})^{I}P_{t}, & b=a+I \\ 0, & others \end{matrix}\right.$$

where $\alpha_{I}\text{=}1/(1-(P_{i})^{I})$, $P_{i}$,$P_{d}$and $P_{t}$ are symbol insertion, deletion and transmission probability, respectively. We initialized these parameters with the observed error rates as shown in Fig. 5C.

In the transition from $x_{j}=a$ to $x_{j+1}=b$, the length of the output sequence $\underline{u}$ is related to both $a$ and $b$. Define $Q_{j,a,b}(\underline{u})=P(\underline{u}|x_{j}=a,x_{j+1}=b,w_{1j}w_{2j})$ as the output probability. The readout base sequence $\underline{u}$ is mapped to the bit pair, $u_{1}$ and $u_{2}$. The watermark bits are $w_{1j}$ and $w_{2j}$. If an insertion error occurs, there are four options for inserted bases (A/T/G/C) and the insertion probability of each base is 1/4. If a base substitution error occurs, the mapped double-layer bits may have two situations:

(a) Two bits are both different, {A(00)C(11), T(01)G(10), C(11)A(00), G(10)T(01)};

(b) Only one bit is different, {A(00)T(01), G(10)C(11), T(01)C(11), A(00)G(10), T(01)A(00), C(11)G(10), C(11)T(01), G(10)A(00)}.

Therefore, the effective substitution error probabilities in the two cases are $\left(\frac{1}{3}P_{f}\right)$ and $\left(\frac{2}{3}P_{f}\right)$, respectively. $P_{f}$ is denoted as $P_{f}=f(1-P_{s})+(1-f)P_{s}$, where $f$ is the average sparsity in the sparse vector set, and $P_{s}$ is the symbol substitution probability.

Then, $Q_{j,a,b}(\underline{u})$ is calculated as

$$Q_{j,a,b}\text{=}\left\{\begin{matrix} \frac{\alpha_{I}(P_{i})^{b-a+1}\left(\frac{1}{4}\right)^{b-a+1}P_{d}+\alpha_{I}(P_{i})^{b-a}\left(\frac{1}{4}\right)^{b-a}P_{t}(1-P_{f})}{P_{a,b}}, u_{1}^{\text{*}}\text{=}w_{1j},u_{2}^{\text{*}}\text{=}w_{2j} \\ \frac{\alpha_{I}(P_{i})^{b-a+1}\left(\frac{1}{4}\right)^{b-a+1}P_{d}+\alpha_{I}(P_{i})^{b-a}\left(\frac{1}{4}\right)^{b-a}P_{t}\left(\frac{2}{3}P_{f}\right)}{P_{a,b}}, \\ \text{ } u_{1}^{\text{*}}\text{=}w_{1j},u_{2}^{\text{*}}\neq w_{2j}\text{ }or\text{ }u_{1}^{\text{*}}\neq w_{1j},u_{2}^{\text{*}}=w_{2j} \\ \frac{\alpha_{I}(P_{i})^{b-a+1}\left(\frac{1}{4}\right)^{b-a+1}P_{d}+\alpha_{I}(P_{i})^{b-a}\left(\frac{1}{4}\right)^{b-a}P_{t}\left(\frac{1}{3}P_{f}\right)}{P_{a,b}}, u_{1}^{\text{*}}\neq w_{1j},u_{2}^{\text{*}}\neq w_{2j} \end{matrix}\right.$$

Here, $\sum_{\underline{u}} Q_{j,a,b}(\underline{u})=1$, $u_{1}^{\text{*}}\text{=}u_{1_{b-a+1}}$is the received bit associated with the pseudo-random bit $w_{1j}$, and $u_{2}^{\text{*}}\text{=}u_{2_{b-a+1}}$is the received bit associated with the pseudo-random bit $w_{2j}$.

**Modified Forward-backward algorithm**

For the readout base sequence $\underline{r}^{0}$, define the forward probability $F_{j}(y)$ as$F_{j}(y)=P(r_{0},\cdot\cdot\cdot,r_{j-1+y},x_{j}=y),\text{ }y\in\text{X}$ and the backward probability $B_{j}(y)$ $B_{j}(y)=P(r_{j+y},\cdot\cdot\cdot|x_{j}=y),\text{ }y\in\text{X}$ [11]. Then, the recursive computation is performed as follows:

Step 1. Initialize the forward and backward a-priori probabilities [4].

Step 2. Calculate the forward and backward probabilities of all states in each position $t_{j}$ [11].

The forward probability of the state $y$ at position index $j$ is calculated as:

.

Similarly,$B_{j}(y)$is calculated as:

$B_{j}(y)=\sum_{b=y-1}^{y+I} B_{j+1}(b)\cdot P_{y,b}\cdot Q_{i,y,b}(r_{j+y},\cdot\cdot\cdot,r_{j+b})$.

Step 3. The offset value corresponding to the maximum probability$F_{j}\left(y\right)B_{j}\left(y\right)$ of each position $t_{j}$ is calculated as:

$$max\_drift_{j}^{ }=\mathrm{argmax}\left(F_{j}(y)B_{j}(y)\right)$$

Step 4. The offset of each base position from the original position, $t_{j+\max\_drift_{j}}$, is used to identify the insertion/deletion.

1. **Comparison between LDPC codes and RS codes**

We compared the LDPC codes with RS codes, considering that RS codes were previously used in DNA data storage [7,13]. For the binary LDPC code applied, the code length *n_1_* is 64,800, the length of information vector *k_1_* is 54,000 and the code rate *R_1_* is 5/6=0.8333. For **a** fair comparison, we chose RS(4985,4154) over the finite field *GF*(2*^p^*), where *p*=13. In binary, *n_2_*=4985×13=64,805, *k_2_*=4154×13=54,002, *R_2_*=0.8333 [6]. This RS code has the closest coding parameters to our LDPC code, for the code length and the symbol size *p* of RS code are constrained.

To compare the error correction capability, we evaluated these two codes using computer simulation with the same error type we actually faced (Fig. 5D). The results are listed in Table S6. This LDPC code can correct all the errors in 10,000 codewords when the input error rate was lower than 2% (1,296 errors). When the input error rate is lower than 0.68% (415 errors), all the errors can be corrected using this RS code. When the error rate increased, the RS code failed. For example, when the error rate is 0.7%, 10,000 out of 10,000 tries failed.

For the complexity comparison, we mainly focus on the decoding complexity. We repeated decoding 1,000 tries and measured the run time of the two decoders, respectively. The run time of the LDPC and RS decoders was 191 seconds and 54. seconds, respectively, on the same personal computer (Intel® core™ i7 2600 CPU @ 3.40GHz). For RS codes, all the operations are on the finite fields, while belief-propagation decoding of the LDPC code is performed on the real field iteratively [6]. Overall, RS codes have some advantages over LDPC codes in decoding complexity, while the decoding algorithm of LDPC codes is parallel in intrinsic, promising potential high-throughput applications in future DNA data storage.

1. **Assembly of the artificial chromosome**

5~6-Kbp DNA building blocks were outsourced to Tsingke Biological Technology. A ~40-Kbp DNA sub-chunks was assembled from 5~6-Kbp DNA building blocks containing overlaps and *NotI* site in both ends [14,15] (Fig. 1A). Eight building blocks were mixed in a single tube and digested by NotI (NEB). The digested DNA was purified using **an** Axygen DNA purification kit (AP-GX-250) and mixed with linear pCC1-His vectors. The vectors were PCR amplified with overhang primer sets 8~13 in Table S6. The mixed DNA was co-transformed to BY4741 using the LiAc-mediated transformation method [16]. After cultivation of 2~5 days at 30℃, the colony with the expected length was screened by colony PCR. The plasmids were extracted from these yeast clones using Magen HiPure BAC DNA Kits with 10 µg/mL Zymolyase-20T pretreated step. Then, these plasmids were introduced into *E. coli* EPI300 by electro-transformation [17]. Next, the plasmids were re-extracted from *E. coli* and digested by NotI. Finally, the plasmids were identified by PFGE with the condition: 0.5× TBE, 1% agarose, 6 v/cm, 0.1-1.5 s switch time, and 14℃ for 16 hours.

To obtain the final artificial chromosome, we mixed six ~40-Kbp DNA sub-chunks equimolarly (about 500 ng each) and digested them by NotI. DNA was then precipitated by the addition of 0.7 volumes of isopropanol and recovered by centrifugation at 15,000× g for 10 min at 4 ºC. DNA pellet was washed with 700 µL fresh 70% ethanol and recentrifuged twice and then resuspended in 20 μL TE (pH 8.0). A linear 10-Kbp YAC backbone pCC1-Ura was PCR amplified with the primer set 16 (Table S6). Mixed 100 ng linear backbone with the 20 μL resuspended DNA and one-step transformed them into BY4741 by yeast protoplast transformation [16]. We identified the correct plasmids first via PCR assay with primer sets 1~7 listed in Table S6. Then the YAC was extracted from yeast (yMH007) and introduced into *E. coli* EPI300 by electroporation [17]. The chromosome was re-isolated from *E. coli* using QIAGEN Plasmid Maxi Kit (Catalog No. 12163). 100 ng sample was digested with Not I and analyzed by PFGE with the condition: 0.5 × TBE, 1% agarose, 6 v/cm, 1-30 s switch time, and 14℃ for 20 hours.

1. **Chromosome extraction**

The method of chromosome extraction from yeast was modified from the protocol of QIAGEN plasmid Mini Kits (Catalog no. 12123). We picked and cultivated a single colony in 5 ml SC-Ura medium at 30℃ overnight and harvested the yeast cells by centrifugation and washed once by 1 mL ddH_2_O. The removal of the cell wall was referenced to [16]. Then, the yeast pellets were resuspended in 0.3 mL of Buffer P1(50 mM Tris·Cl, pH 8.0; 10 mM EDTA; 100 μg/mL RNase A). The cells should be resuspended completely by vortexing or pipetting up and down until no cell clumps remain. Added 0.3 ml Buffer P2 (200 mM NaOH, 1% SDS), mixed thoroughly by vigorously inverting the sealed tube 4–6 times, and incubated at room temperature (15–25°C) for 5 min. Then added 0.3 mL of chilled Buffer P3 (3.0 M potassium acetate, pH 5.5), mixed immediately and thoroughly by vigorously inverting 4–6 times, and incubated on ice for 5 min. Next, centrifuged at 12000× g in a microcentrifuge for 10 min. Removed supernatant containing plasmid DNA promptly to a new 1.5 ml microtube without using the pipettor. DNA was then precipitated by the addition of 0.7× volume of isopropanol. The precipitated DNA was recovered using centrifugation at 12000× g for 10 min at 4 ºC. DNA pellets were washed with 70% ethanol and resuspended in 20 μL TE (pH 8.0). To reduce interfering reads during sequencing, the extracted chromosome was introduced into *E. coli* EPI300 by electroporation [17] and re-isolated from *E. coli* using QIAGEN Plasmid Maxi Kit (Catalog No. 12163)

1. **Library preparation for nanopore sequencing**

The extracted artificial chromosome was valued by nanodrop and PFGE to ensure the quality of the DNA library. We took 50 fmole DNA for sequencing library preparation following the official protocol with ONT rapid sequencing kit SQK-RAD004 on an ice bath. In particular, the chromosome was rapidly fragmented with only 1 µL Fragmentation Mix (from the kit) and the proper amount of ddH_2_O to make the total reaction volume to 10 µL.

1. **Data readout using nanopore sequencer and proposed processes**

We used MinION sequencer with flow cell R9.4.1 to read bases and MinKNOW for base calling and writing files into fastq format. We added a preprocessing step to polish the very noisy sequencing reads. The polishing process mainly includes two steps. The first is to assemble a coarse chromosome. Then, the reads are mapped and used to polish the coarse chromosome ring.

Specifically, the recovery process from the fastq files is demonstrated as follows.

(1) Long noisy reads are mapped and assembled using Minimap and Miniasm [18].

(2) The coarsely assembled contigs were polished using RACON [19].

(3) The data DNA sequences were located according to ARS positions and the backbone sequence. We identified the most possible locations and lengths of all the known sequences by computing the minimum edit distance.

(4) Insertions and deletions were identified using the modified forward-backward algorithm [11,20].

(5) The substitution errors were corrected using LDPC codes or non-binary LDPC codes on the parallel iterative algorithms [21]. Then, the decoding results are converted into the original image and video files.

1. **Data recovery process using Illumina sequencing data**

The specific procedure using **Illumina** sequencing data is presented in Fig. S14. Steps are as following:

(1) Remove the reads from the host genome and choose a proper number of reads with the predetermined coverage [22].

(2) Choose the optimal k-mer value and assemble the paired-end reads into contigs [22].

(3) Identify the six data sequences according to the ARSs and the vector (or the embedded watermarks), and then obtain the data sequences.

(4) Correct the residual errors in the data sequences using LDPC codes and recover the original files.

**
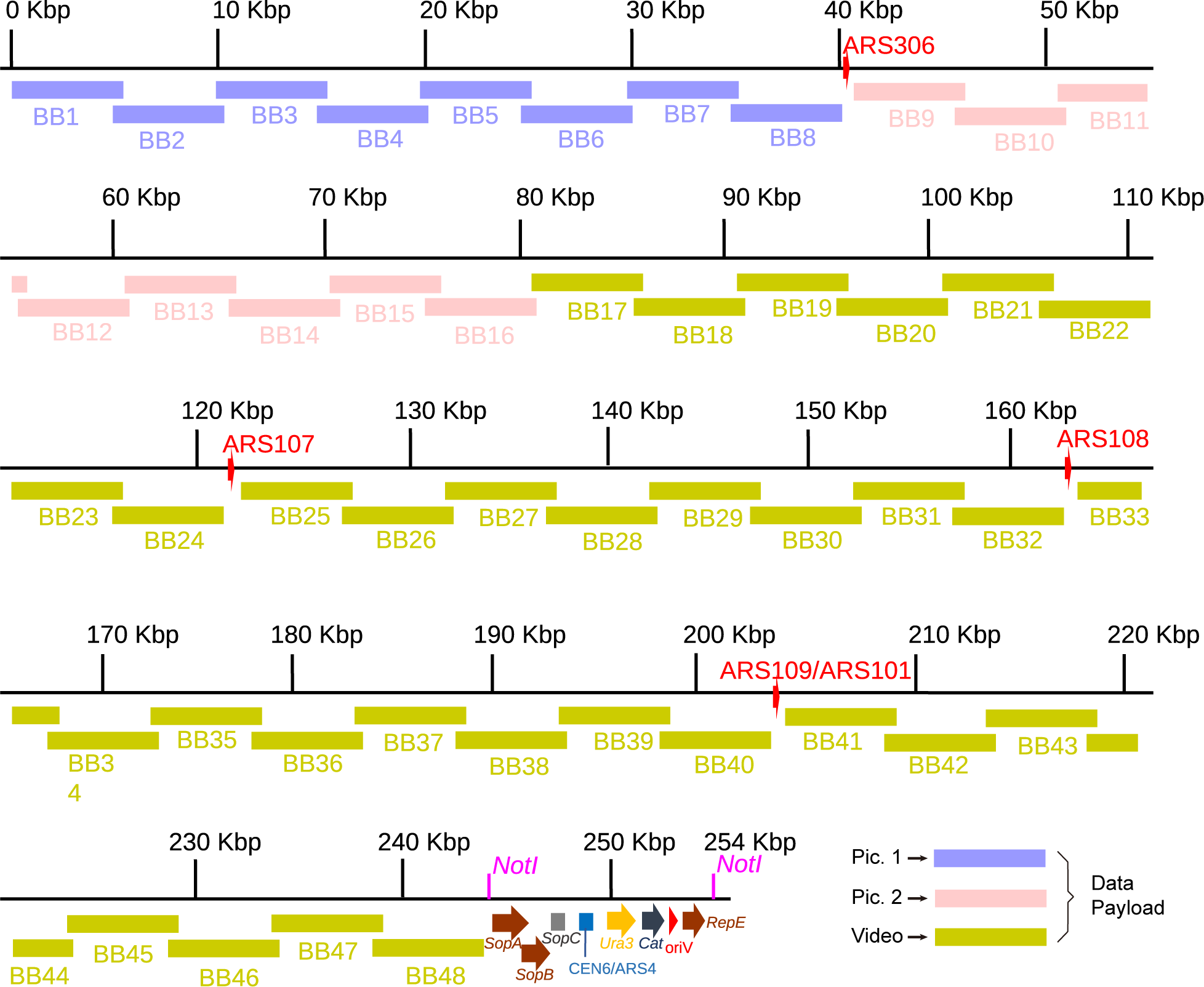
**

**Figure S1.** **Map of the artificial data-carrying chromosome.**

95.27% of the chromosome carries digital data. BB (number) indicates 5~6-Kb building blocks; BB1-BB8, picture 1; BB9-BB16, picture 2; BB17-BB48, video clip. Four additional ARSs were embedded denoted as red arrows. The approximate 10-Kb sequence between the two *NotI* sites is the pCC1-Ura vector which contains elements of *sopA, sopB*, sopC, *RepE,* *Cat*, CEN6/ARS4 and *Ura3*.

**
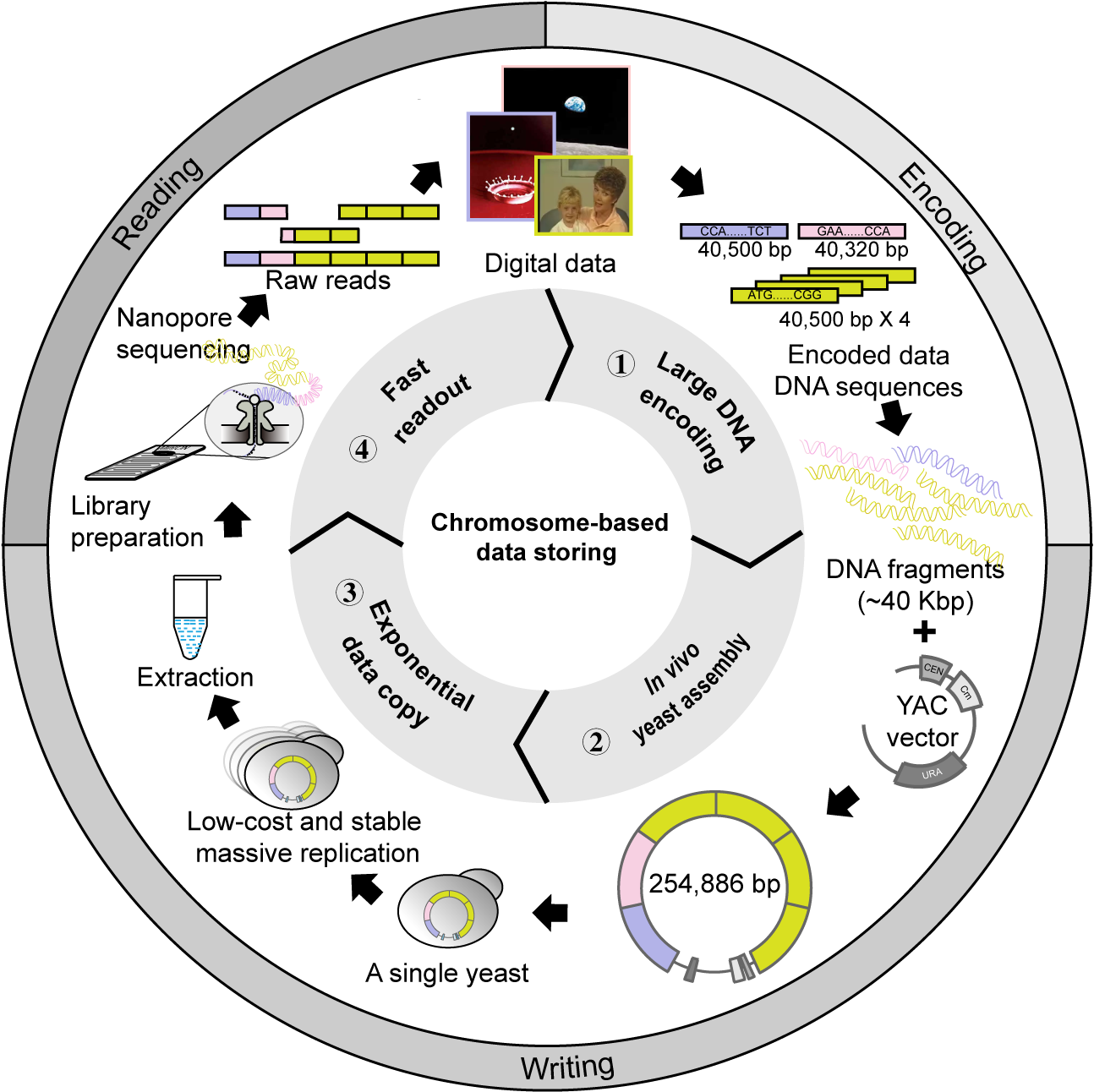
**

**Figure S2.** **Workflow of digital data storage using artificial chromosome.**

**
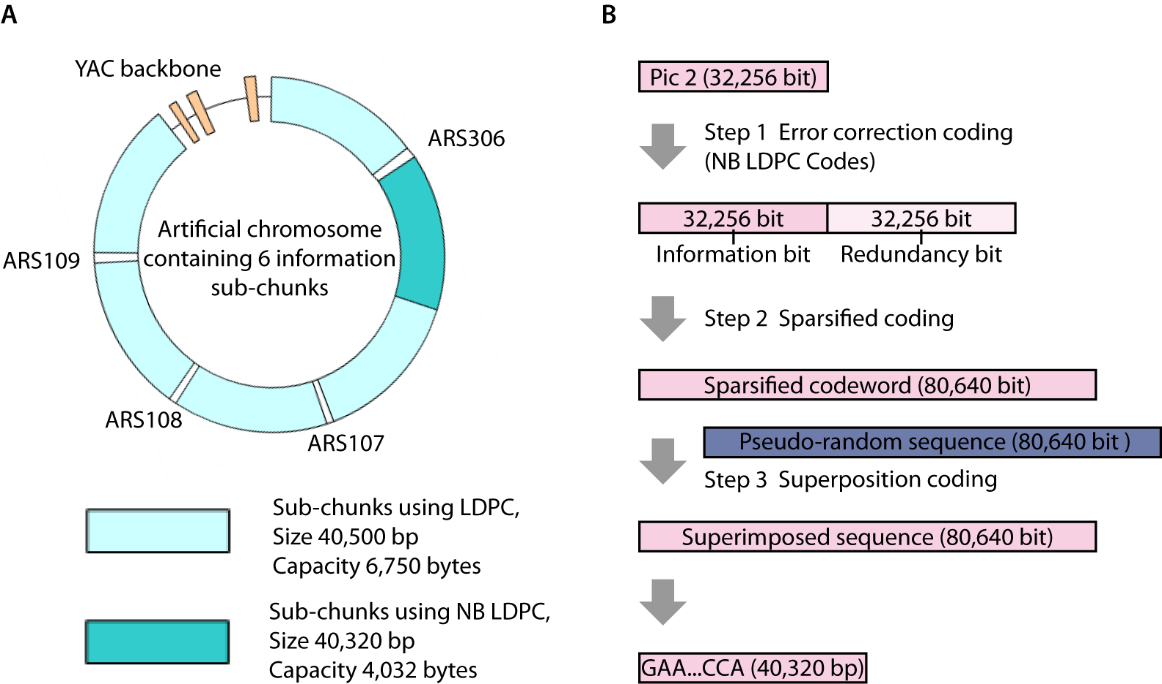
**

**Figure S3. Information sub-chunks and encoding scheme using NB LDPC code.**

**(A),** Two error correction codes with different coding rates were chosen to support the different levels of error tolerance capabilities. **(B),** The encoding method using NB LDPC code.

**
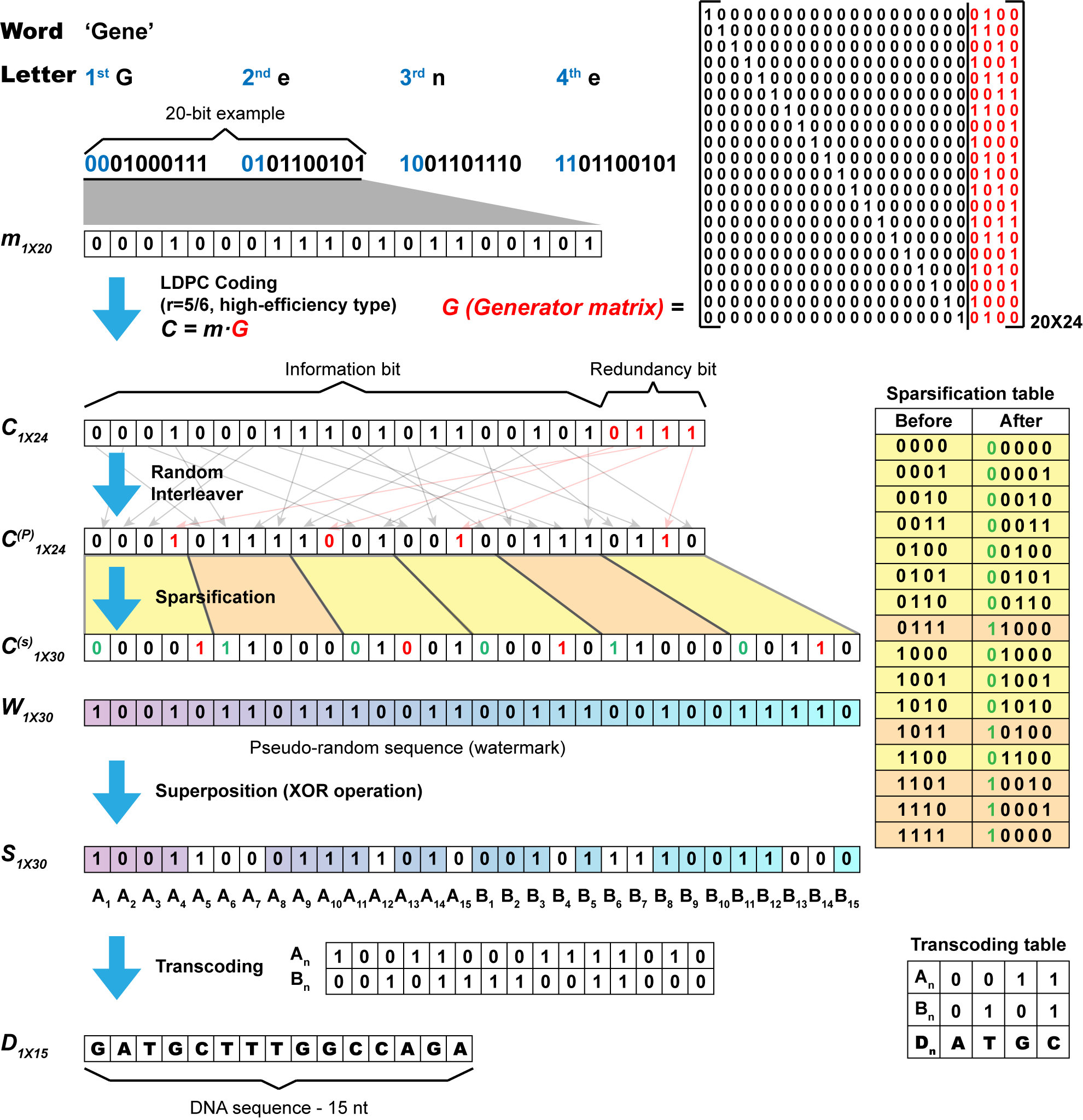
**

**Figure S4. An example of encoding scheme.**

We illustrated the process by encoding a piece of 20-bit message into **a** DNA sequence. First, the bit group of 20 bits, denoted as ***m***, is encoded into the 24-bit LDPC codeword **(though very short, represent the LDPC code)** by ***C***=***m*******G***. Then, the codeword is randomly interleaved. Next, sparsify the interleaved codeword group by group. Each group has four bits, which are mapped into 5 bits according to the sparsification table. The sparsified codeword (30 bit) is superimposed with a predetermined pseudo-random sequence (called watermark). Finally, the superimposed bit sequence is transcoded into DNA sequences (15 nt) according to the transcoding table.

**
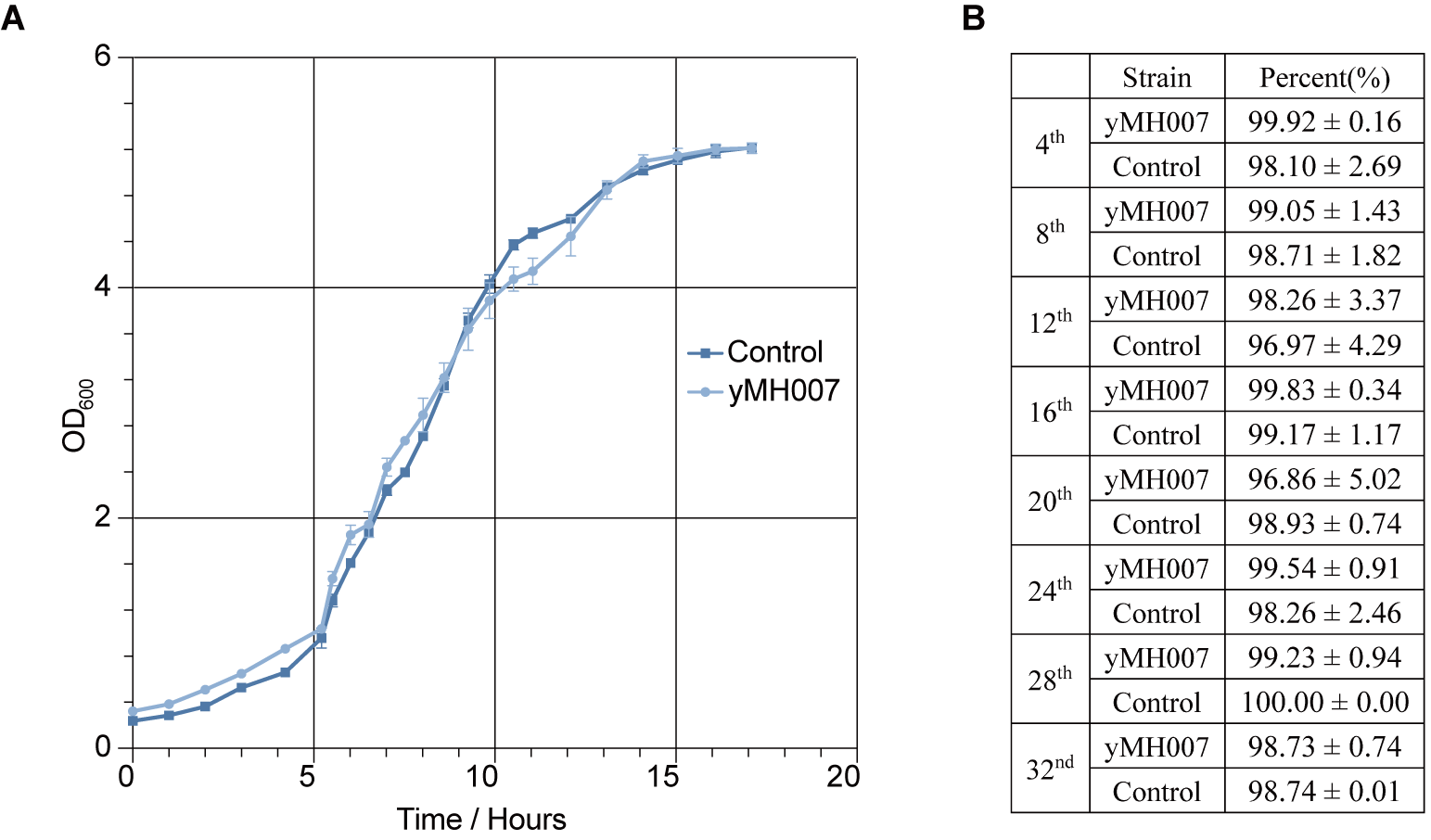
**

**Figure S5. Growth curves and the percentage of data-carrying strain yMH007.**

**(A),** Growth curves of yMH007 and control. Doubling **times are** 2.7 ± 0.1 and 2.6 ± 0.1 hour, respectively. (α=0.05). Results were obtained from three independent experiments. **(B),** Percentage of the data-carrying chromosome in yMH007 and control serial batch-cultured in SC-Ura liquid medium. Data represent the mean ± SD.

**
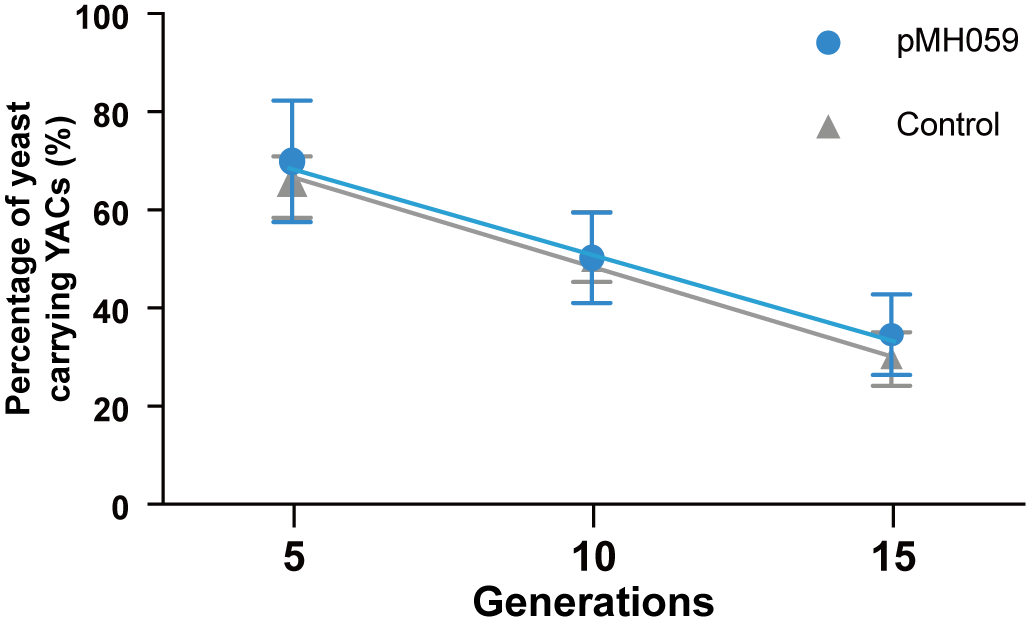
**

**Figure S6. Loss frequency (LF) test in SC medium appended uracil.**

The **LFs of pMH059 and pCC1-Ura are** 7.0% ± 0.3% and 8.6% ± 2.7% (α=0.05), respectively. The LF was calculated from LF= -Δ(ln*Vt*)/Δ*Gt* [23], where *Vt* is the frequency of chromosome-carrying yeast and *Gt* is the number of generations.

**
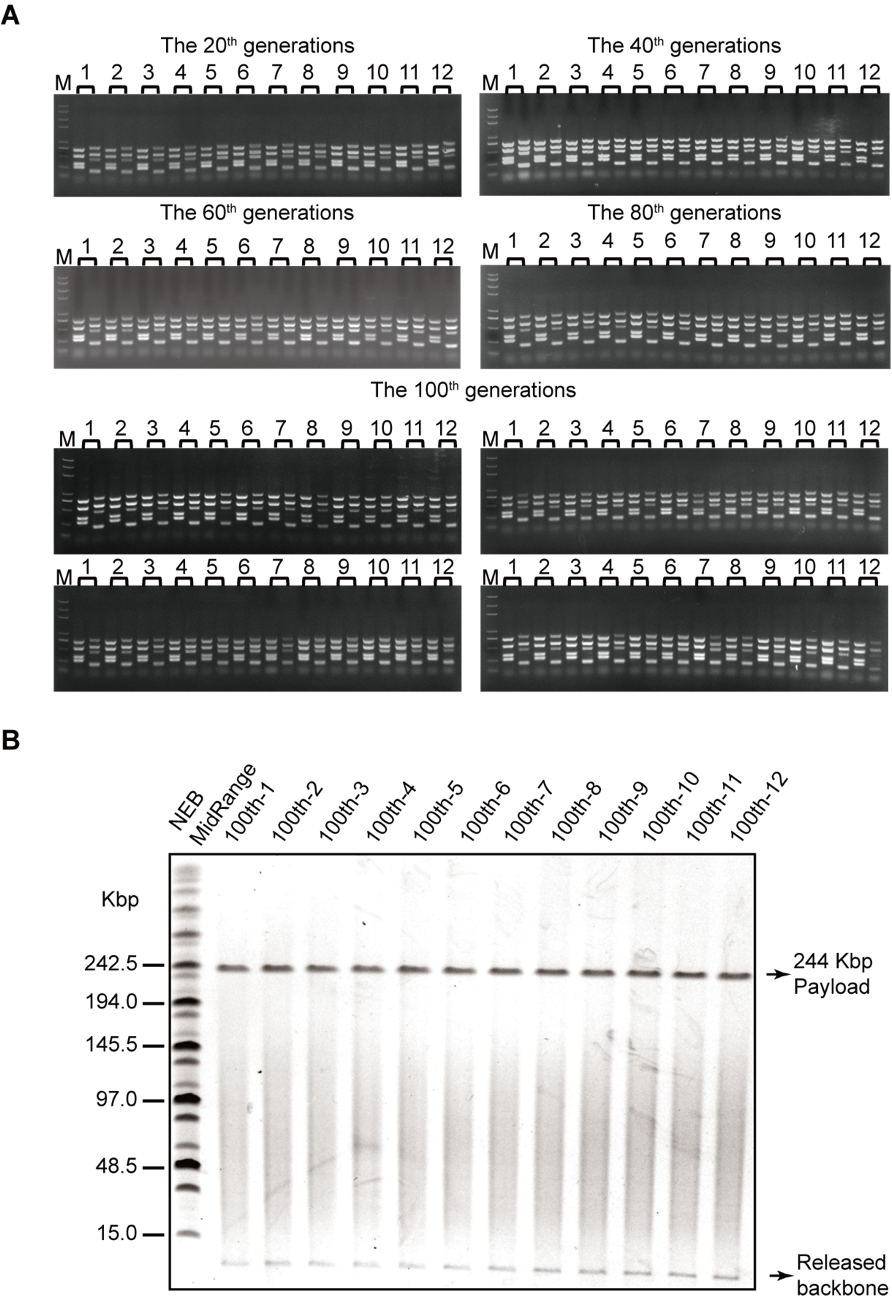
**

**Figure S7. Fidelity assay by Multiplex PCR and PFGE.**

**(A),** Multiplex PCR assay with 20-generation intervals. Eight bands per two lanes represented one colony multiplex PCR result. **Band sizes of the two lanes are** 768 bp, 563 bp, 350 bp, 280 bp, and 831 bp, 605 bp, 466 bp, 206 bp. **(B),** PFGE assay for testing the integrity of 12 data-carrying chromosomes at the 100^th^ generation

**
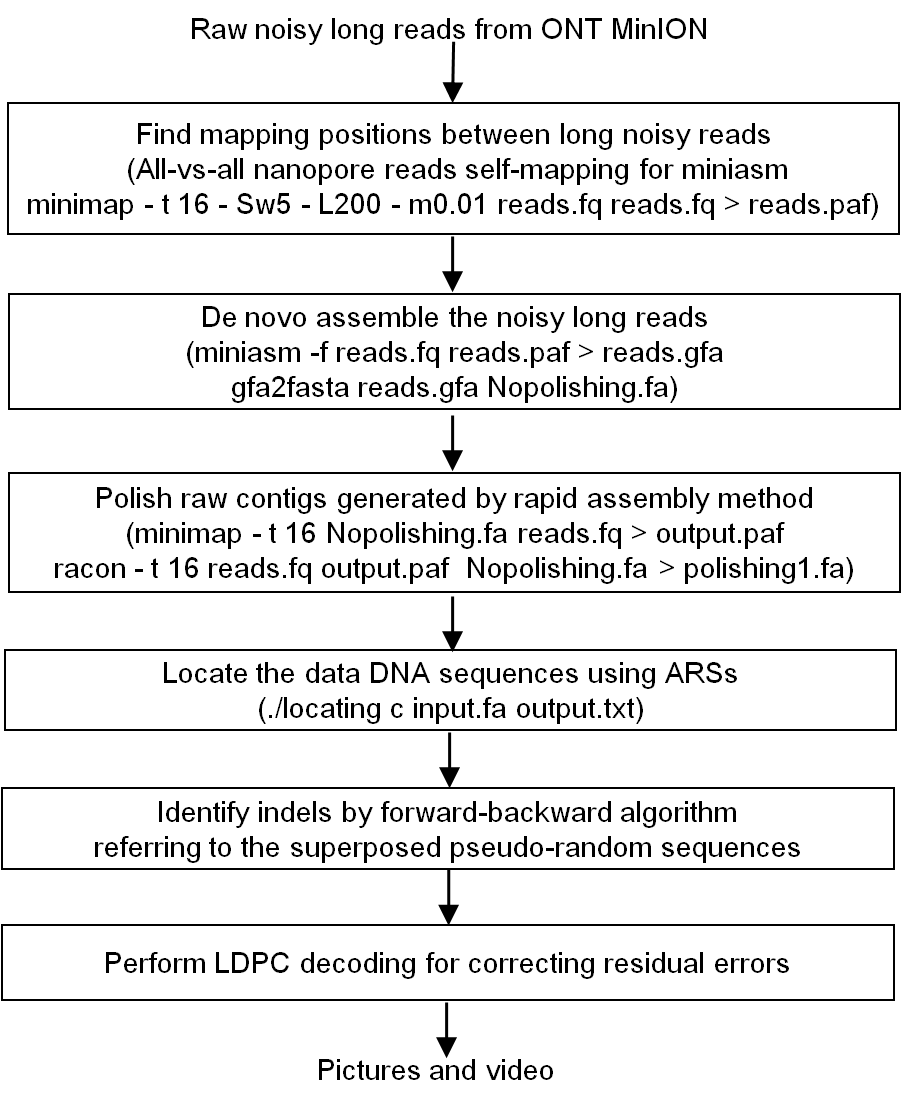
**

**Figure S8. Flow diagram of data recovery from ONT sequencing reads.**

**
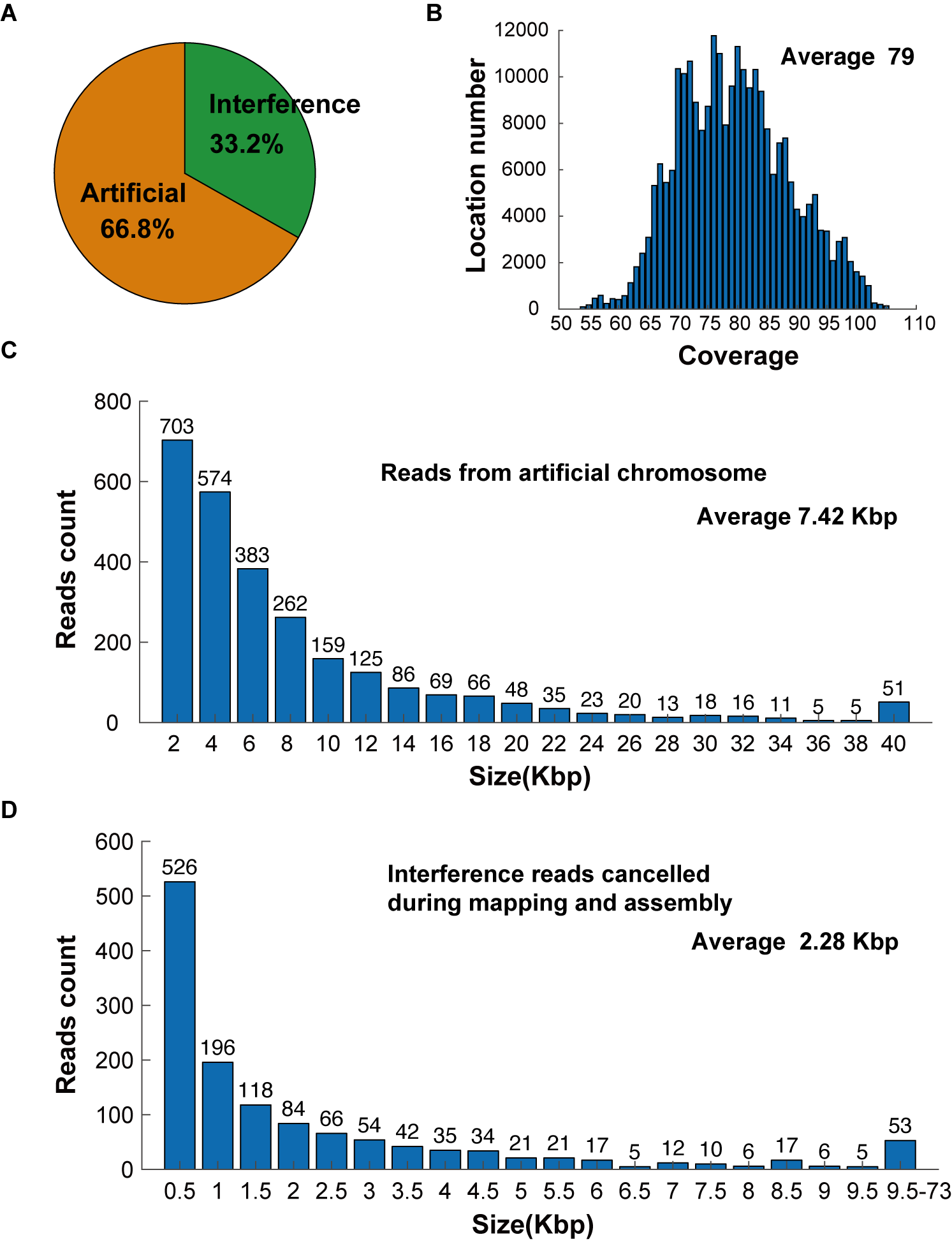
**

**Figure S9. Distribution of sequencing reads.**

**(A),** The ratio of data-carrying chromosome reads **in** 4000 sequencing reads. There remained some reads from the host genome or the unlinked adapters, which were viewed as interference. **(B),** Distribution of the coverage. **(C), Length** distribution of the reads from the artificial chromosome. **(D),** Length distribution of the interference reads

| Head | ARS306 |
| --- | --- |
|  |  |
| ARS107 | ARS108 |
|  |  |
| ARS109 | Tail |
|  |  |

**Figure S10. Boundary identification of data sequence.**

All the boundaries of data sub-chunks were identified using ARSs, according to the sliding computation of the edit distance, in which indels and substitutions were all included. All the curves were generated using one assembly with an error rate of 0.53%.

**
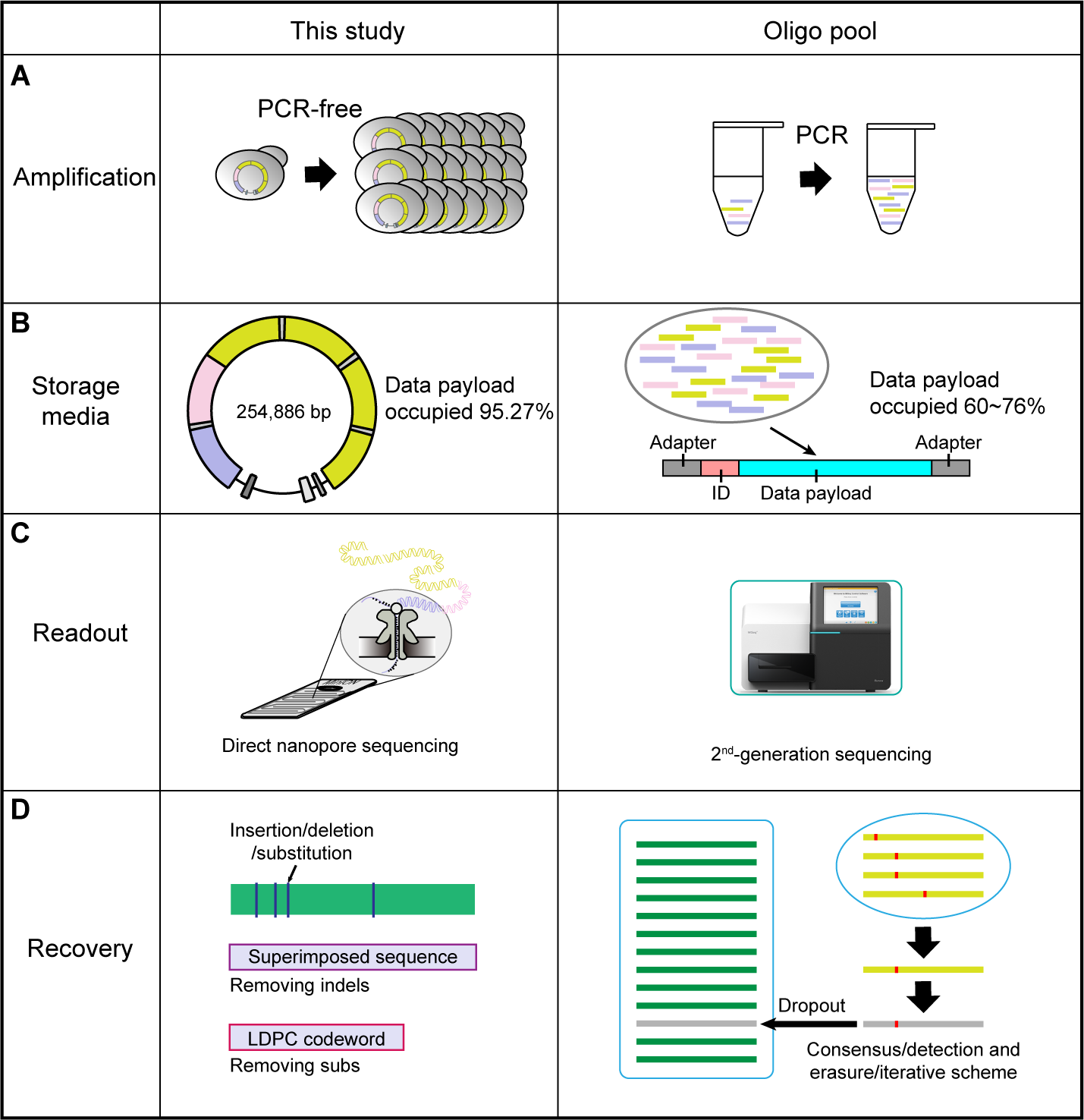
**

**Figure S11. Comparison of chromosome-based and oligo-based DNA storage.**

**(A),** Amplification using yeast replication is PCR-free, showing a lower error rate and amplification bias (Fig. S12), requiring less cost. **(B),** This study using large watermarked and encoded DNA achieves a relatively high logic density (Table S2) but the production of the first version (‘the master’) is expensive. **(C),** The large chromosome can be fast readout using ONT MinION sequencer. **(D),** Our encoding method is robust to **tolerate** severe indels and compatible with the noisy output reads of the nanopore sequencers.


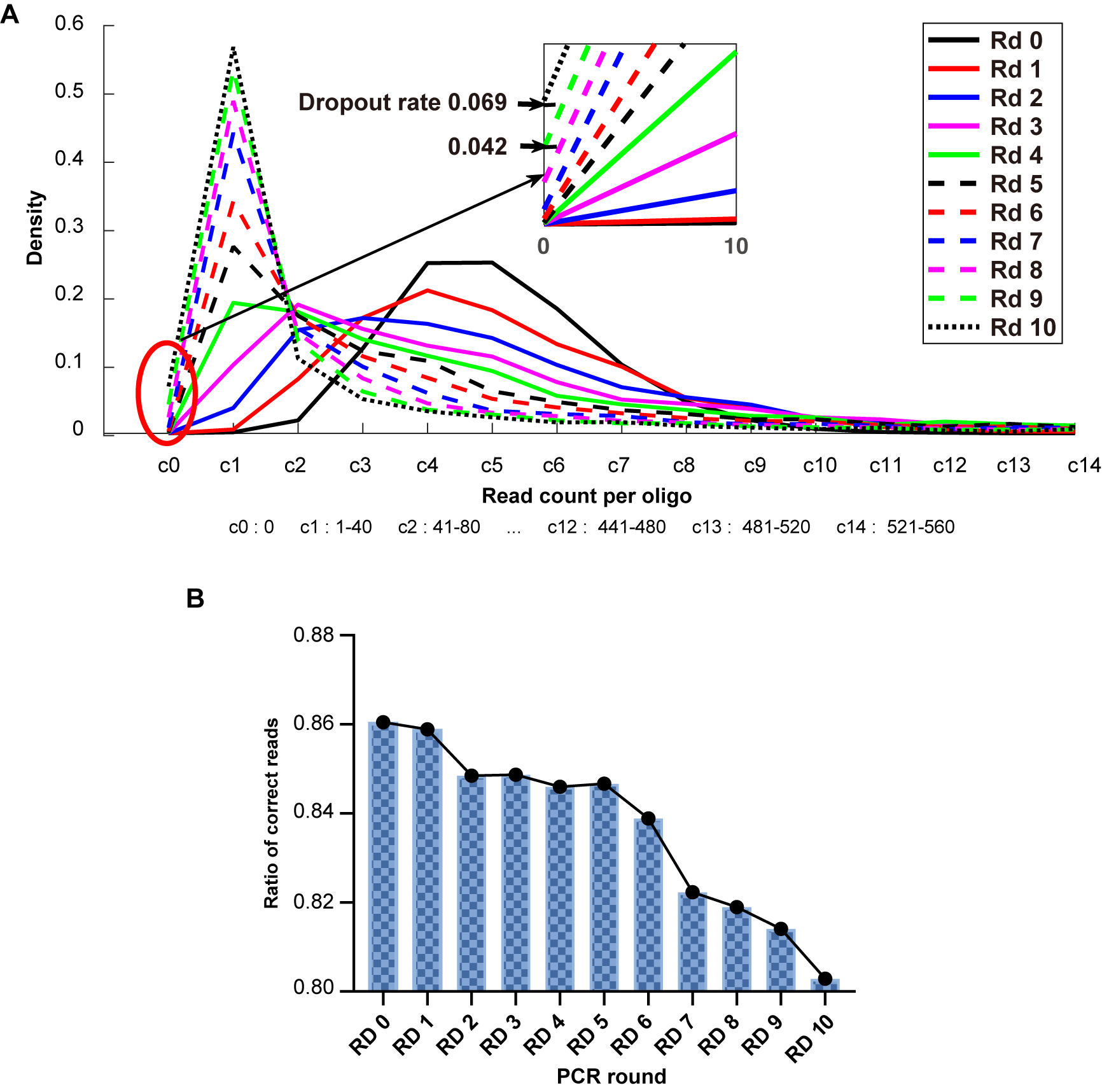


**Figure S12. Oligo-pool-based data copy via multiple rounds of PCRs.**

**(A),** The bias of PCR-based amplification. The data sequences were decomposed into an oligo pool (2698 149-nt oligos), amplified by 10 rounds of PCR, each round including 10 cycles, and then sequenced by **Illumina** platform, according to a previous protocol [2]. With the increasing of the PCR rounds, the bias became significant and a small fraction of oligos drop out. **(B),** The ratio of correct reads in different rounds of PCR.

**
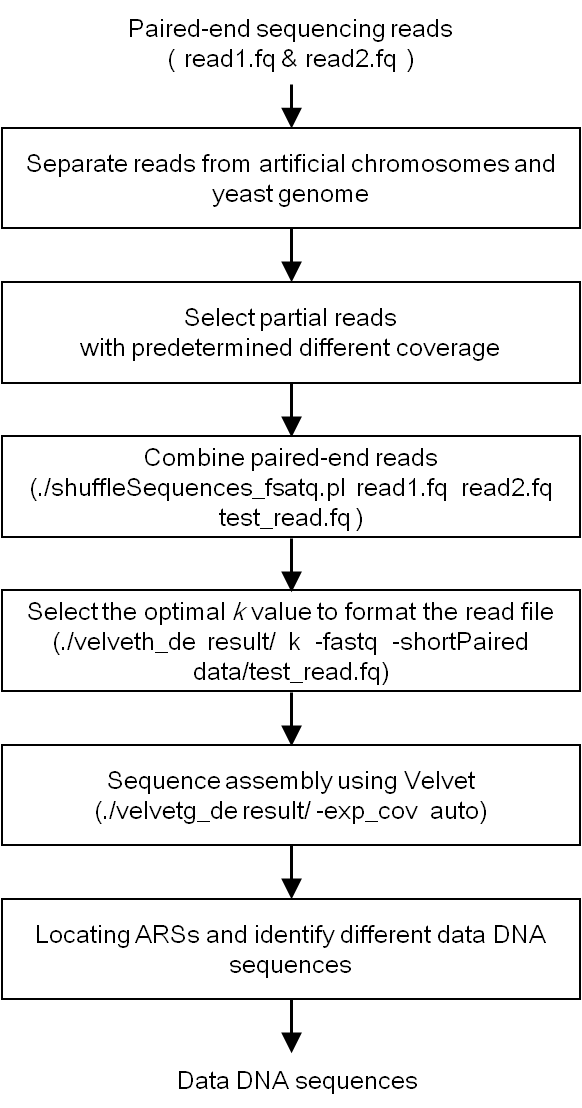
**

**Figure S13. Flow diagram of data recovery from Illumina sequencing reads.**


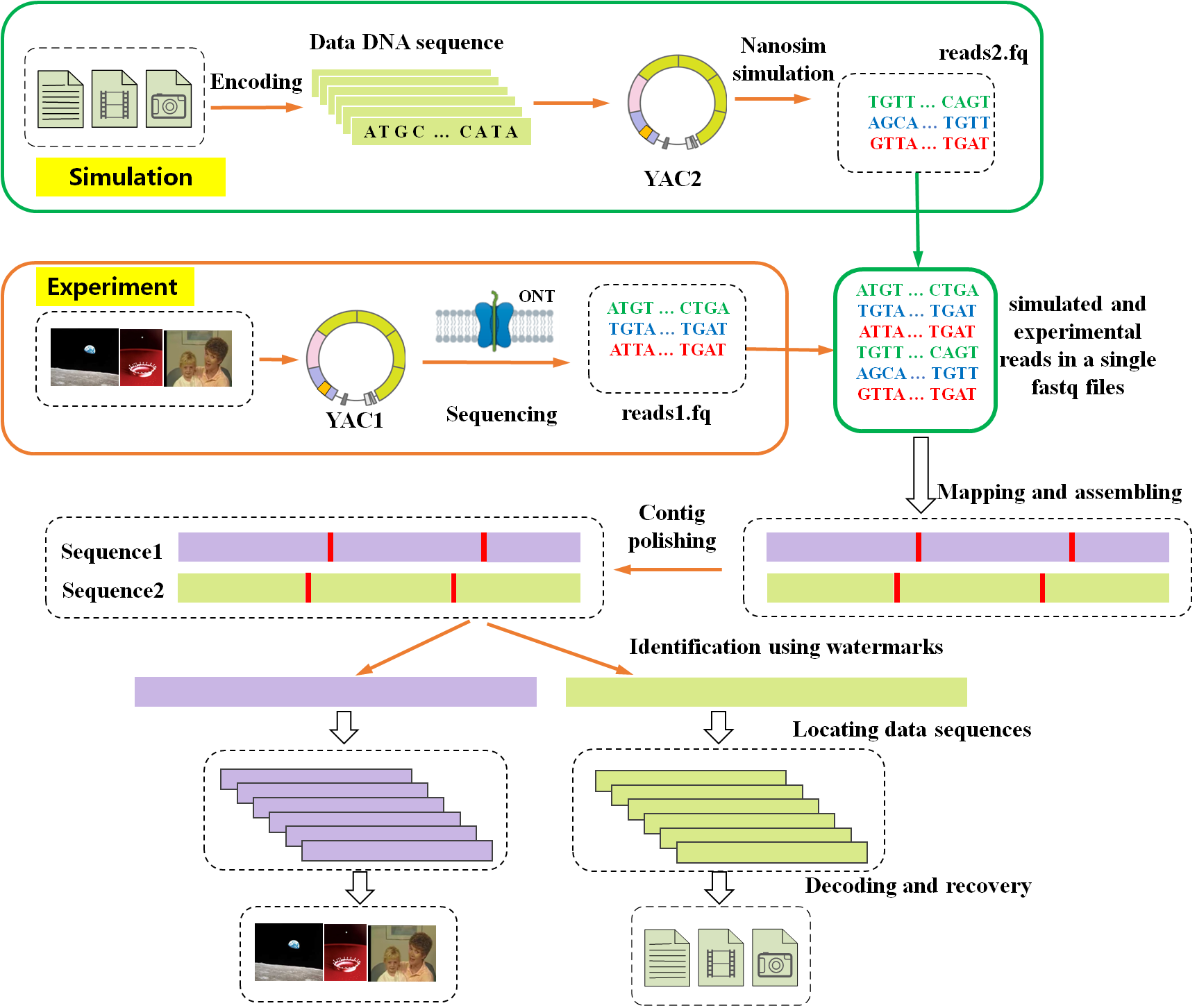


**Figure S14. Simulation of parallel readout of two chromosomes.**

A parallel readout of two YACs was carried out, within which one is real and the other is made by simulation. We encoded the data with another watermark and assembled a virtual chromosome YAC2. This YAC contained the same vector and ARSs sequence as the real one did. We generated sequencing reads of YAC2 using Nanosim [24]. We merged the reads from the simulation of YAC2 and the real sequencing reads from YAC1. Following the same recovery process, we discriminated the two contigs using the embedded watermark and retrieved the original files.

**Table S1. Comparison of data encoded *in vivo* to prior work.**

| Ref. | Bits encoded | Encoded base pairs x Number | Storage medium |
| --- | --- | --- | --- |
| Davis [25] | 35 | 28 x 1 | Plasmid/*E. coli* |
| Bancroft *et a*l. [26] | 561 | 232 x 1  247 x 1 | Plasmid/*E. coli* |
| Wong *et al*. [27] | 1,106 | 57-99 x 7 | Plasmid/*E. coli* |
| Gustafsson *et a*l. [28] | 1,007 | 800 x 1 | Plasmid/*E. coli* |
| Yachie *et al*. [29] | 124/128 | 62/64 x 1 | Plasmid/*E. coli*  *B. subtilis* genome |
| Ailenberg *et al*. [30] | 1,688 | 844 x 1 | Plasmid/*E. coli* |
| Gibson *et al*. [17] | 7,920 | 1,081-1,246 x 4 | *Mycoplasma* genome watermarks |
| Shipman *et al*. [31] | 30,781 | 58 x 732 | *E. coli* genome  CRISPR array |
| Nguyen *et al*. [32] | 16,368 | 372 x 22 | Plasmid/*E. coli* |
| Hao *et al*. [33] | 3,645,440 | 155 x 11520 | Plasmid/*E. coli* |
| This study | 302,256 | **242,820 x 1** | Artificial chromosome/  *S. cerevisiae* |

**Table S2. Comparison of logic density to prior work.**

| Ref. | Bits per base including primers (vectors) | Bits per base excluding primers (vectors) |
| --- | --- | --- |
| Church *et al.* [34] | 0.60 | 0.83 |
| Goldman *et al*. [35] | 0.19 | 0.29 |
| Grass *et al*. [36] | 0.83 | 1.16 |
| Bornholt *et al*. [37] | 0.57 | 0.85 |
| Erlich *et al*. [2] | 1.19 | 1.57 |
| Blawat *et al*. [38] | 0.89 | 1.08 |
| Organic *et al*. [7] | 0.81 | 1.10 |
| This study | 1.19 | 1.24 |

**Table S3. Information of files stored in the designed artificial chromosome.**

| **File name** | **Size**  **(Byte)** | **File**  **type** | **Encoding** | **Data DNA sequence** |
| --- | --- | --- | --- | --- |
| 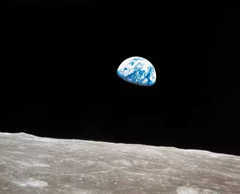  Earth rise | 4,029 | jpg | Non-binary LDPC (64512,32256)  Converted into 1 codeword  +pseudo-random sequence |  |
| Text file | 3 | txt |
| 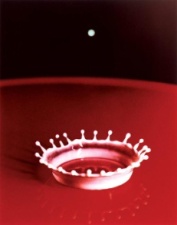  Milk Drop Coronet | 6,624 | jpg | Binary LDPC (64800,54000)  Converted into 1 codeword  +pseudo-random sequence |  |
| Text file | 126 | txt |
| 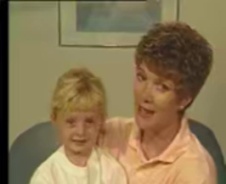  Mother and Daughter | 26,092 | mp4 | Binary LDPC (64800,54000)  Converted into 4 codewords  +pseudo-random sequence |  |
| Text file | 908 | txt |
|  | 37,782 | jpg, mp4, txt |  | **242,820 bases** |

**Table S4. Recovery of data payload using 2^nd^-generation sequencing reads**

| Sequencing reads | Reads count | Coverage | Data payload  error rate | Error rate  after recovery |
| --- | --- | --- | --- | --- |
| G20  (150 nt) | 22000 | 25.9 | 0.001388 | 0 |
|  | 23000 | 27.1 | 0.000696 | 0 |
|  | 25000 | 29.4 | 0.002425 | 0 |
|  | 31000 | 36.5 | 0.012891 | 0 |
|  | 32000 | 37.7 | 0 | 0 |
|  | 33000 | 38.8 | 0 | 0 |
|  | 35000 | 41.2 | 0 | 0 |
| G40  (150 nt) | 20000 | 23.5 | 0.009740 | 0 |
|  | 23000 | 27.1 | 0.001771 | 0 |
|  | 25000 | 29.4 | 0.005963 | 0 |
|  | 30000 | 35.3 | 0.000264 | 0 |
|  | 32000 | 37.7 | 0 | 0 |
|  | 33000 | 38.8 | 0 | 0 |
|  | 35000 | 41.2 | 0 | 0 |
| G60  (150 nt) | 30000 | 35.3 | 0.000350 | 0 |
|  | 35000 | 41.2 | 0.000202 | 0 |
|  | 40000 | 47.1 | 0.000531 | 0 |
|  | 45000 | 53.0 | 0.000531 | 0 |
|  | 50000 | 58.8 | 0.000531 | 0 |
|  | 55000 | 64.73 | 0.000531 | 0 |
|  | 60000 | 70.62 | 0 | 0 |
| G80  (150 nt) | 23000 | 27.1 | 0.000683 | 0 |
|  | 25000 | 29.4 | 0.000597 | 0 |
|  | 30000 | 35.3 | 0.000601 | 0 |
|  | 35000 | 41.2 | 0.000226 | 0 |
|  | 38000 | 44.7 | 0.000272 | 0 |
|  | 40000 | 47.1 | 0 | 0 |
|  | 45000 | 53.0 | 0 | 0 |
| G100  (150 nt) | 24000 | 28.2 | 0.001137 | 0 |
|  | 26000 | 30.6 | 0.001034 | 0 |
|  | 28000 | 33.0 | 0.000815 | 0 |
|  | 35000 | 41.2 | 0.000079 | 0 |
|  | 38000 | 44.7 | 0 | 0 |
|  | 40000 | 47.1 | 0 | 0 |

*The read assembly was carried out with Velvet [22].

**Table S5. Simulation of LDPC and RS codes under the same error type***

| **Input Error rate** | **Failures in 10,000 codewords** | | |
| --- | --- | --- | --- |
|  | LDPC(64512, 32256), *R*=1/2 | LDPC(64800, 54000), *R*=5/6=0.83 | RS(4985,4154) over *GF*(2^13^),  RS(64805, 54002) in binary,  R=54002/64805=0.83 |
| 0.5% | 0 | 0 | 0 |
| 0.6% | 0 | 0 | 0 |
| 0.64% | 0 | 0 | 0 |
| 0.7% | 0 | 0 | 10,000 |
| 1% | 0 | 0 | 10,000 |
| 2% | 0 | 0 | 10,000 |
| 2.5% | 0 | 1 | 10,000 |
| 3.0% | 0 | 128 | 10,000 |
| 6% | 0 | 10,000 | 10,000 |
| 7% | 3 | 10,000 | 10,000 |
| 8% | 9 | 10,000 | 10,000 |

* The error type is random substitution, according to the error property after the indel correction (Fig. 5D). All the errors are scattered throughout the whole codeword.

**Table S6. Yeast used in this work.**

| Strain | Characteristics |
| --- | --- |
| BY4741 | *MATa his3Δ1 leu2Δ0 met15Δ0 ura3Δ0* |
| yMH001 | BY4741/pCC1-His-Pic1 |
| yMH002 | BY4741/pCC1-His-Pic2 |
| yMH003 | BY4741/pCC1-His-Vd1 |
| yMH004 | BY4741/pCC1-His-Vd2 |
| yMH005 | BY4741/pCC1-His-Vd3 |
| yMH006 | BY4741/pCC1-His-Vd4 |
| yMH007 | BY4741/Artificial chromosome |
| yMH008-yMH019 | 20th-generation of yMH007 |
| yMH020-yMH031 | 40th-generation of yMH007 |
| yMH032-yMH043 | 60th-generation of yMH007 |
| yMH044-yMH055 | 80th-generation of yMH007 |
| yMH056-yMH103 | 100th-generation of yMH007 |
| yMH104 | BY4741/pCC1-Ura |

**Table S7. Primers used in this work.**

|  | **Fragment** | **Primer** | **Sequence** |
| --- | --- | --- | --- |
| 1 | Verify-1 | Forward | TCAGGAGTACGAACGCCATCGACTTACCA |
|  |  | Reverse | TGACCTCGGAATGTCTACCCCGGGA |
| 2 | Verify-2 | Forward | AAAGGCTCACACGCACCATCCT |
|  |  | Reverse | TCGTGTCTGGTGGGAAATCACAGGCA |
| 3 | Verify-3 | Forward | TAGATACGACCCACCGCCGGCCA |
|  |  | Reverse | TAGAGAGTGCCGTGGTGCACTGACG |
| 4 | Verify-4 | Forward | TTTGAGTCACTACCTTGCAGCCAAGAAGGTAT |
|  |  | Reverse | TATTGCGTACCCAGTAGTTAGGCCGATTCCT |
| 5 | Verify-5 | Forward | AGCTCGTGTCAAGAAACACACAAACAATCTCCACA |
|  |  | Reverse | TGCATACCCTTACTCTTCGCTTCACCTGCT |
| 6 | Verify-6 | Forward | AATCCGCTTACTACGGCAGGCTCTCA |
|  |  | Reverse | ACTTTCCGCATCCATAATCGAGATTGCGAT |
| 7 | Verify-7 | Forward | TACTGGAGGCCGAGCAGCACTAGAGGA |
|  |  | Reverse | TCCGGGCTGTCATCATTAAACTGTGCAATG |
| 8 | pCC1-Pic1 | Forward | agcacatcgctgaggtatcattacttcatgagataaattaacctgcagggcggccgcGGCGTAATCATGGTCATAGCTGTTTCCTGTGT |
|  |  | Reverse | gtactggtagctggggtatcgcctgaatgacggcacaagcggaaggacagcgggtgggcggccgcACTGGCCGTCGTTTTACAACGTCG |
| 9 | pCC1-Pic2 | Forward | gtacagagggcagaacacctcaggccactcgtgctgatgctttcgaccagcggccgcGGCGTAATCATGGTCATAGCTGTTTCCTGTGT |
|  |  | Reverse | tgaaaaaagtaggagacaacactttccttcctcacgaagaagttaagctcctgcagggcggccgcACTGGCCGTCGTTTTACAACGTCG |
| 10 | pCC1-Vd1 | Forward | gcatttagttaggaggacacgaagacccccggtcttgaagctattaatggcggccgcGGCGTAATCATGGTCATAGCTGTTTCCTGTGT |
|  |  | Reverse | gagagttgcgacgaaaaagagcaccgctcggtttcttgacgatacgagtacattcatgcggccgcACTGGCCGTCGTTTTACAACGTCG |
| 11 | pCC1-Vd2 | Forward | acatcgtcatgaatcgctggttaagcaggggaggttgaccgatcactctgcggccgcGGCGTAATCATGGTCATAGCTGTTTCCTGTGT |
|  |  | Reverse | ccaggaatcgcttcccacattgtagattgcacattacggacgcgagttctatgagtcgcggccgcACTGGCCGTCGTTTTACAACGTCG |
| 12 | pCC1-Vd3 | Forward | tgaccggtattgactgcgggggcccaatgcaagctggagagattatgccgcggccgcGGCGTAATCATGGTCATAGCTGTTTCCTGTGT |
|  |  | Reverse | atgccaagtctgtctgtgtggagtccgacagatctacccccgtgttaggttgattcagcggccgcACTGGCCGTCGTTTTACAACGTCG |
| 13 | pCC1-Vd4 | Forward | tacaaatcctcgggcctacaggctaaccaagatatggacccgagtccgggcggccgcGGCGTAATCATGGTCATAGCTGTTTCCTGTGT |
|  |  | Reverse | taggcaccatacctacgtctgtgaagtctttatacgctggcacaagacccgctaggggcggccgcACTGGCCGTCGTTTTACAACGTCG |
| 14 | Linker-up | Forward | AAGCTAGGTGACGACAGCGGGATAGA |
|  |  | Reverse | GACGATACGAGTACATTCATTGGTCGAAAGCATCAGCACGAGTGG |
| 15 | Linker-down | Forward | CGTGCTGATGCTTTCGACCAATGAATGTACTCGTATCGTCAAGAAACCGAGC |
|  |  | Reverse | CCAGGCTCCCGGATGAACGCA |
| 16 | pCC1-244K | Forward | ctacaaatcctcgggcctacaggctaaccaagatatggacccgagtccgggcggccgcGGTATGTGCTCTTCTTATCTCCTTTTGTAGT |
|  |  | Reverse | tggtactggtagctggggtatcgcctgaatgacggcacaagcggaaggacagcgggtgggcggccgcGGTCGGTCATCCGGATATAGTT |
| 17 | MultiPCR-1 | Forward | CTGGACTCTACTGATGTCTGGACA |
|  |  | Reverse | AGAGCGCTATCTCAGAGCAT |
| 18 | MultiPCR-2 | Forward | GCGACGGATTCACTTCTCCAA |
|  |  | Reverse | CGATGGAGTAGTCGTTAACGGTTA |
| 19 | MultiPCR-3 | Forward | GATGGTCGCTAGTGCAACAAGA |
|  |  | Reverse | GAAGGTGGAGGAGGAATGGT |
| 20 | MultiPCR-4 | Forward | CAATGCCTACTGCCGCACTA |
|  |  | Reverse | GCAGGTGATCCAGCATCACAT |
| 21 | MultiPCR-5 | Forward | CATCTGCAGCACAGATAGCTCA |
|  |  | Reverse | GAGTAAGACGACCGGTGCTAA |
| 22 | MultiPCR-6 | Forward | CATCGCAGTTCTAATGCGATTGA |
|  |  | Reverse | CCGCGGACGTAACACAATCA |
| 23 | MultiPCR-7 | Forward | CCATCGCGAGGTATCACGTT |
|  |  | Reverse | CATCCGGCTCAAGGTACATGAA |
| 24 | MultiPCR-8 | Forward | CGACTGCTACCTCACAACTTGA |
|  |  | Reverse | CGGAAGGCACCGTATTGTAGATGAA |

**References**

1. Gibson DG, Benders GA, Andrews-Pfannkoch C*, et al.* Complete chemical synthesis, assembly, and cloning of a Mycoplasma genitalium genome. *Science*. 2008; **319**(5867): 1215-20.

2. Erlich Y, Zielinski D. DNA Fountain enables a robust and efficient storage architecture. *Science*. 2017; **355**(6328): 950-3.

3. Richardson TJ, Urbanke RL. The capacity of low-density parity-check codes under message-passing decoding. *IEEE Trans Inf Theory*. 2001; **47**(2): 599-618.

4. Richardson TJ, Shokrollahi MA, Urbanke RL. Design of capacity-approaching irregular low-density parity-check codes. *IEEE Trans Inf Theory*. 2001; **47**(2): 619-37.

5. Tomek KJ, Volkel K, Simpson A*, et al.* Driving the Scalability of DNA-Based Information Storage Systems. *Acs Synth Biol*. 2019; **8**(6): 1241-8.

6. Lin S, Costello DJ. *Error control coding*: Prentice hall; 2001.

7. Organick L, Ang SD, Chen YJ*, et al.* Random access in large-scale DNA data storage. *Nat Biotechnol*. 2018; **36**(7): 242-8.

8. Holmes JK. *Spread spectrum systems for GNSS and wireless communications*: Artech House Norwood; 2007.

9. Golomb SW. *Shift register sequences*: Aegean Park Press; 1967.

10. Mercier H, Bhargava VK, Tarokh V. A survey of error-correcting codes for channels with symbol synchronization errors. *IEEE Commun Surv Tutor*. 2010; **12**(1): 87-96.

11. Davey MC, MacKay DJ. Reliable communication over channels with insertions, deletions, and substitutions. *IEEE Trans Inf Theory*. 2001; **47**(2): 687-98.

12. Rabiner L, Juang B. An introduction to hidden Markov models. *IEEE ASSP Mag*. 1986; **3**(1): 4-16.

13. Grass RN, Heckel R, Puddu M*, et al.* Robust chemical preservation of digital information on DNA in silica with error‐correcting codes. *Angew Chem Int Ed*. 2015; **54**(8): 2552-5.

14. Wu Y, Li B-Z, Zhao M*, et al.* Bug mapping and fitness testing of chemically synthesized chromosome X. *Science*. 2017; **355**(6329): eaaf4706.

15. Xie Z-X, Li B-Z, Mitchell LA*, et al.* “Perfect” designer chromosome V and behavior of a ring derivative. *Science*. 2017; **355**(6329): eaaf4704.

16. Kouprina N, Larionov V. Selective isolation of genomic loci from complex genomes by transformation-associated recombination cloning in the yeast Saccharomyces cerevisiae. *Nat Protoc*. 2008; **3**(3): 371-7.

17. Gibson DG, Glass JI, Lartigue C*, et al.* Creation of a bacterial cell controlled by a chemically synthesized genome. *Science*. 2010; **329**(5987): 52-6.

18. Li H. Minimap and miniasm: fast mapping and de novo assembly for noisy long sequences. *Bioinformatics*. 2016; **32**(14): 2103-10.

19. Vaser R, Sović I, Nagarajan N*, et al.* Fast and accurate de novo genome assembly from long uncorrected reads. *Genome Res*. 2017; **27**(5): 737-46.

20. Liu Y, Chen W. Decoding on adaptively pruned trellis for correcting synchronization errors. *China Commun*. 2017; **14**(7): 1-9.

21. Richardson T, Kudekar S. Design of Low-Density Parity Check Codes for 5G New Radio. *IEEE Commun Mag*. 2018; **56**(3): 28-34.

22. Zerbino DR, Birney E. Velvet: algorithms for de novo short read assembly using de Bruijn graphs. *Genome Res*. 2008; **18**(5): 821-9.

23. Løbner-Olesen A, Atlung T, Rasmussen K. Stability and replication control of Escherichia coli minichromosomes. *J Bacteriol*. 1987; **169**(6): 2835-42.

24. Yang C, Chu J, Warren RL*, et al.* NanoSim: nanopore sequence read simulator based on statistical characterization. *GigaScience*. 2017; **6**(4): gix010.

25. Davis J. Microvenus. *Art J*. 1996; **55**(1): 70-4.

26. Bancroft C, Bowler T, Bloom B*, et al.* Long-term storage of information in DNA. *Science*. 2001; **293**(5536): 1763-5.

27. Wong PC, Wong KK, Foote H. Organic data memory using the DNA approach. *Commun Acm*. 2003; **46**(1): 95-8.

28. Gustafsson C. For anyone who ever said there's no such thing as a poetic gene. *Nature*. 2009; **458**(7239): 703.

29. Yachie N, Sekiyama K, Sugahara J*, et al.* Alignment‐based approach for durable data storage into living organisms. *Biotechnol Prog*. 2007; **23**(2): 501-5.

30. Ailenberg M, Rotstein OD. An improved Huffman coding method for archiving text, images, and music characters in DNA. *Biotechniques*. 2009; **47**(3): 747-51.

31. Shipman SL, Nivala J, Macklis JD*, et al.* CRISPR–Cas encoding of a digital movie into the genomes of a population of living bacteria. *Nature*. 2017; **547**(7663): 345-9.

32. Nguyen HH, Park J, Park SJ*, et al.* Long-term stability and integrity of plasmid-based DNA data storage. *Polymers*. 2018; **10**(1): 28.

33. Hao M, Qiao H, Gao Y*, et al.* A mixed culture of bacterial cells enables an economic DNA storage on a large scale. *Commun Biol*. 2020; **3**(1): 416.

34. Church GM, Gao Y, Kosuri S. Next-generation digital information storage in DNA. *Science*. 2012; **337**(6102): 1628.

35. Goldman N, Bertone P, Chen SY*, et al.* Towards practical, high-capacity, low-maintenance information storage in synthesized DNA. *Nature*. 2013; **494**(7435): 77-80.

36. Grass RN, Heckel R, Puddu M*, et al.* Robust Chemical Preservation of Digital Information on DNA in Silica with Error-Correcting Codes. *Angew Chem Int Ed*. 2015; **54**(8): 2552-5.

37. Bornholt J, Lopez R, Carmean DM*, et al.* Toward a DNA-Based Archival Storage System. *IEEE Micro*. 2017; **37**(3): 98-104.

38. Blawat M, Gaedke K, Huetter I*, et al.* Forward error correction for DNA data storage. *Procedia Comput Sci*. 2016; **80**: 1011-22.
